# Decompose quantitative susceptibility mapping (QSM) to sub-voxel diamagnetic and paramagnetic components based on gradient-echo MRI data

**DOI:** 10.1016/j.neuroimage.2021.118477

**Published:** 2021-08-14

**Authors:** Jingjia Chen, Nan-Jie Gong, Khallil Taverna Chaim, Maria Concepción García Otaduy, Chunlei Liu

**Affiliations:** a Department of Electrical Engineering and Computer Sciences, University of California, Berkeley, Berkeley, CA, USA; b Vector Lab for Intelligent Medical Imaging and Neural Engineering, International Innovation Center of Tsinghua University, Shanghai, China; c LIM44, Instituto e Departamento de Radiologia, Faculdade de Medicina, Universidade de Sao Paulo, Sao Paulo, Brazil; d Helen Wills Neuroscience Institute, University of California, Berkeley, Berkeley, CA, USA

**Keywords:** Susceptibility, QSM, Decompose, Sub-voxel, Paramagnetic, Diamagnetic, Gradient-recalled echo

## Abstract

**Purpose:**

A method named DECOMPOSE-QSM is developed to decompose bulk susceptibility measured with QSM into sub-voxel paramagnetic and diamagnetic components based on a three-pool complex signal model.

**Methods:**

Multi-echo gradient echo signal is modeled as a summation of three weighted exponentials corresponding to three types of susceptibility sources: reference susceptibility, diamagnetic and paramagnetic susceptibility relative to the reference. Paramagnetic component susceptibility (PCS) and diamagnetic component susceptibility (DCS) maps are constructed to represent the sub-voxel compartments by solving for linear and nonlinear parameters in the model.

**Results:**

Numerical forward simulation and phantom validation confirmed the ability of DECOMPOSE-QSM to separate the mixture of paramagnetic and diamagnetic components. The PCS obtained from temperature-variant brainstem imaging follows the Curie’s Law, which further validated the model and the solver. Initial in vivo investigation of human brain images showed the ability to extract sub-voxel PCS and DCS sources that produce visually enhanced contrast between brain structures comparing to threshold QSM.

## 1. Introduction

Quantitative Susceptibility Mapping (QSM) is a magnetic resonance imaging (MRI) technique to non-invasively quantify tissue magnetic susceptibility (Bilgic et al., 2012; Deistung et al., 2017; Li et al., 2011; Liu et al., 2015b, 2015a; Wang and Liu, 2015). Tissue susceptibility change is involved in normal aging and many disease developments in the brain. QSM has shown superior contrast and potential utilities in revealing iron level alternation in brain aging processes (Betts et al., 2016; Bilgic et al., 2012; Zhang et al., 2018), imaging myelination during brain development (Argyridis et al., 2014; Zhang et al., 2018) a imaging protein accumulations in Alzheimer’s disease (AD) (Gong et al., 2019), uncovering dysmyelination (C. Liu et al., 2011)and demyelination in multiple sclerosis (MS) (Chen et al., 2013; Wang et al., 2019; Wisnieff et al., 2015; Zhang et al., 2016), enhancing contrast of calcifications (Deistung et al., 2013b; Straub et al., 2017; Wang et al., 2020) as well as developing biomarker for Parkinson’s Disease (PD) diagnosis (Barbosa et al., 2015; Guan et al., 2019, 2017a, 2017b; He et al., 2015; Murakami et al., 2015; Sjöström et al., 2017).

QSM, however, does not characterize the sub-voxel susceptibility distribution. With the limited resolution (~1 mm) of MRI, a mixture of paramagnetic and diamagnetic susceptibility sources (at molecular scales) may exist in one voxel. The frequency contribution from the opposing susceptibility components may cancel each other in part or in whole, resulting in the total QSM to decrease or appear near zero. For instance, deep brain nuclei contain both paramagnetic iron and diamagnetic myelin (Curnes et al., 1988; Hametner et al., 2018; Stüber et al., 2014); fibrotic livers commonly contain both paramagnetic iron and diamagnetic collagens (Arezzini et al., 2003; Philippe et al., 2007; Wei et al., 2020); kidney inflammation and fibrosis contain both paramagnetic iron and diamagnetic collagens (Xie et al., 2013); *β*-amyloid may colocalize with iron in AD brains (Bousejra-ElGarah et al., 2011; Derry et al., 2020; Lee et al., 2004). Therefore, the ability to separate the opposing susceptibility sources at the sub-voxel level will provide more specific quantification of the magnetic properties of tissue.

There have been a few attempts to separate the opposing susceptibility sources (Lee et al., 2017; Schweser et al., 2011). Lee et al. used both *R*_2_ and $R_{2}^{*}$ measurements to obtain the estimation of $R_{2}^{\prime }$. $R_{2}^{\prime }$ is considered to be linearly affected by absolute susceptibilities, while frequency shift is modeled as a linear composition of susceptibilities convolving with the magnetic dipole kernel. Additionally, MEDI regularization (T. Liu et al., 2011) was used to reduce artifacts. In Schweser et al.’s work, both $R_{2}^{*}$ and bulk susceptibility χ are assumed to depend linearly on concentration of iron, concentration of myelin and a constant term. The coefficients are pre-calculated from postmortem study and magnetization transfer saturation (MTS) images. These models assume that voxel-average magnetic susceptibility is the linear summation of paramagnetic and diamagnetic susceptibility. However, the fundamental signal progression of a multi-echo gradient echo sequence (GRE) involves complex exponentials. While the linear approximation holds when the phase accumulation is small, in general, this is not the case as it has been shown that the phase evolves nonlinearly as a function of echo time in many brain regions (Cronin et al., 2017; Sood et al., 2017).

Hereby, we model the problem with three-pool complex exponentials and develop an algorithm to estimate the opposing susceptibility sources within a voxel using solely multi-echo GRE data. We refer the method as DiamagnEtic COMponent and Paramagnetic cOmponent SEparation or DECOMPOSE-QSM. Numerical forward field simulations, phantom experiments and ex vivo temperature-dependent GRE scans are used to validate the proposed method. The work has been partially presented at the 2021 Annual Meeting of the International Society of Magnetic Resonance in Medicine (Chen et al., 2021a, 2021b).

## 2. Theory

**Fig. 1.**
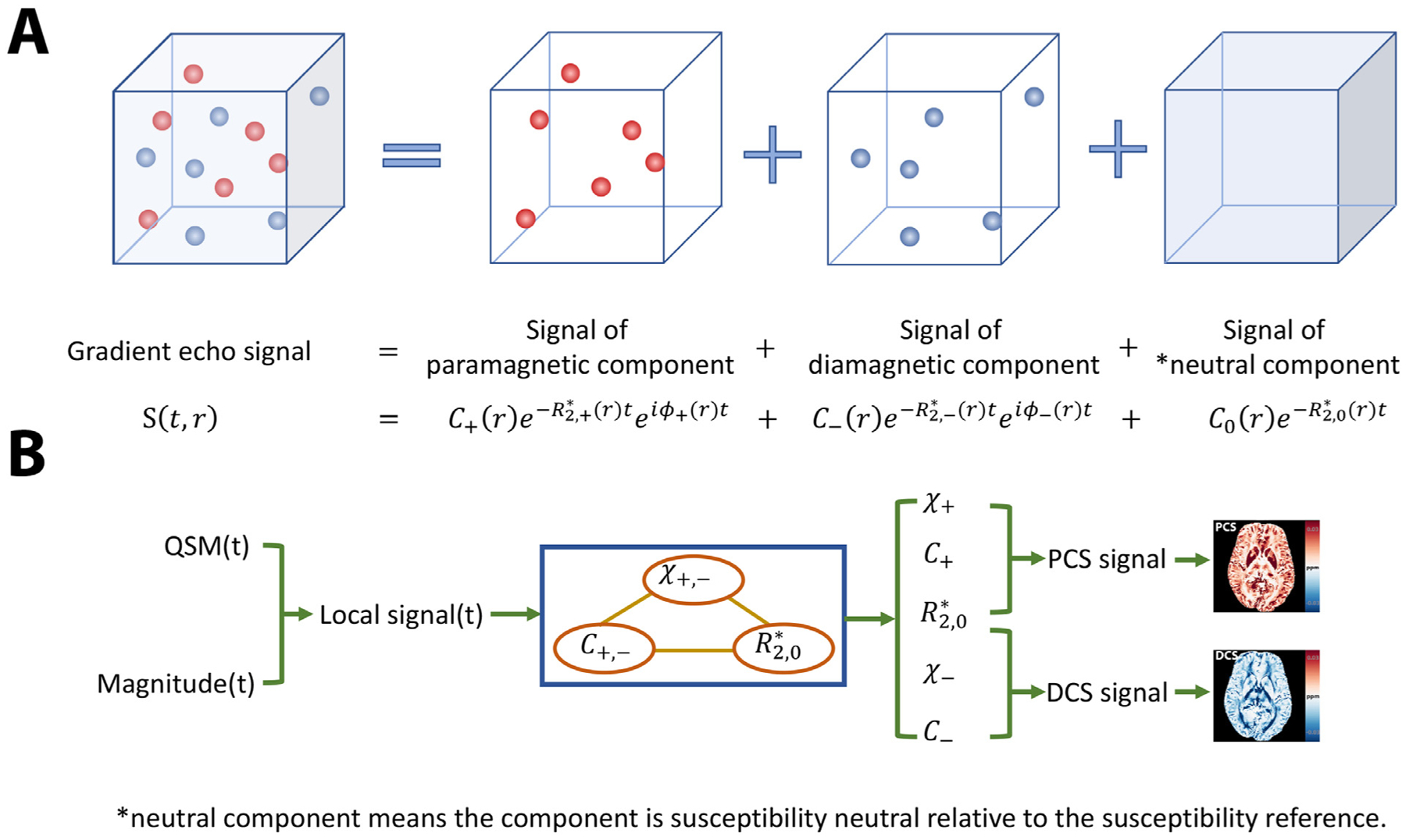
A cartoon illustration of the signal model and the scheme of the solver. (A)Signal of a voxel with mixture of paramagnetic and diamagnetic sources can be decomposed into three pools of signal contributions. The signal outside the susceptibility sources has zero phase. (B) A flowchart of the proposed algorithm. The algorithm takes inputs of echo-time-dependent QSM and Magnitude to compose the local signal. The proposed alternating direction solver processes the local signal and outputs the estimated unknowns. With the estimated parameters, maps of paramagnetic component susceptibility (PCS) and diamagnetic component susceptibility (DCS) are constructed respectively.

DECOMPOSE-QSM is based on a 3-pool signal model. Each voxel is considered to be composed of three distributed pools of magnetic sources: paramagnetic, diamagnetic and magnetically “neutral” (with respect to the reference susceptibility of the imaging volume) components (Fig. 1A). Paramagnetic and diamagnetic sources are modeled as spheres that produce dipole fields outside the spheres but contain uniform magnetization within. The GRE signal of each pool is characterized by a complex exponential with its magnitude following an exponential $R_{2}^{*}\text{-decay }$ and its frequency shift proportional to its magnetic susceptibility after the magnetic field contribution from outside the voxel is deconvolved. For the neutral pool, the frequency shift is zero. The total GRE signal of the voxel is a weighted summation of these three pools.

### 2.1. Three-pool signal model

The sub-voxel structure in Fig. 1A illustrates the complex signals originating from multiple compartments. There are 3 components in such a voxel: the paramagnetic component with volume susceptibility χ_+_ and transverse relaxation rate $R_{2,+}^{*}$, the diamagnetic component with volume susceptibility *χ*_−_ and transverse relaxation rate $R_{2,-}^{*}$, and the reference susceptibility medium with volume susceptibility *χ*_0_ = 0 and transverse relaxation rate $R_{2,0}^{*}$. The reference susceptibility is generally the mean susceptibility within the field of view dominated by water. As being derived in Appendix 1, transverse relaxation rate is a linear function of corresponding susceptibility at the static dephasing regime (Brown, 1961; Yablonskiy and Haacke, 1994),

(1) $$R_{2,+,-}^{*}=a\left|\chi_{+,-}\right|+R_{2,0}^{*},$$

where $a=\frac{2\pi }{9\sqrt{3}}\gamma B_{0}$ (details are in Appendix 1) that evaluated at 3T is 323.5 Hz/ppm. The intercept of the linear approximation of $R_{2}^{*}(\chi )$ corresponds to the transverse relaxation rate of the reference susceptibility medium $R_{2,0}^{*}$.

The susceptibility sources are initially modeled to be spherical. Later, we show that the specific geometry has little influence on the components susceptibility we define. For the phase of each susceptibility source, as long as the majority of the dipole field is within the voxel, the total field perturbation of the voxel will predominantly come from the interiors of the spheres as the exterior fields cancel out due to the symmetrical dipole field distribution (Appendix 2). Thus, the field perturbation contributing from the inside of the sphere is then $B_{z,in}=\frac{2}{3}\chi B_{0}$, where χ is the volume susceptibility of the source and *B*_0_ is the external static field strength.

Therefore, the total GRE signal *S*(*t*) of the voxel with the three components can be written as

(2) $$\text{S}\left(t;C_{+},C_{-},C_{0},\chi_{+},\chi_{-},R_{2,0}^{*}\right)\;=C_{+}e^{-\left(a\chi_{+}+R_{2,0}^{*}+i\;\frac{2}{3}\;\chi_{+}\gamma B0\right)t}\;+C_{-}e^{-\left(-a\chi_{-}+R_{2,0}^{*}+i\;\frac{2}{3}\;\chi_{-}\;\gamma B0\right)t}+C_{0}e^{-R_{2,0}^{*}t},$$

with *C*_+_, *C*_−_, *C*_0_ indicating the concentrations of the corresponding components.

### 2.2. Algorithm design

To estimate the unknowns (*C*_+_, *C*_−_, *C*_0_, χ_+_, χ_−_, $R_{2,0}^{*}$), we solve the following optimization problem,

(3) $$\underset{C_{+},C_{-},C_{0},\chi_{+},\chi_{-},R_{2,0}^{*}}{\text{min}}f\left(S\left(C_{+},C_{-},C_{0},\chi_{+},\chi_{-},R_{2,0}^{*};t\right),y(t)\right)$$

where *y*(*t*) is the measured multi-echo complex signal and *f*(·) is the objective function. We define *y*(*t*) and *f*(·) as follows.

The raw signal of a voxel contains phase contribution from sources outside the voxel while our signal model in Eq. (2) contains only sub-voxel contributions. To ensure that *y*(*t*) is consistent with our model, rather than using the raw signal, we synthesize a local signal with magnitude M(*t*) as follows

(4) $$y(t)=\text{M}(t)e^{i\phi_{in}}=\text{M}(t)e^{-i\;\frac{2}{3}\;\chi (t)\gamma B_{0}t},$$

where M(*t*) is the magnitude of the raw signal. Note that QSM of each echo, *χ*(*t*), is used instead of the average QSM across all echoes. The reason is that with the susceptibility sources being a mixture, the phase evolution is bound to be echo-time dependent (Cronin et al., 2017; Sood et al., 2016). By using the QSM to synthesize the signal, we eliminate background phase contribution from outside the voxel.

The parameters to be estimated can be categorized into two classes: nonlinear parameters (*χ*_−_, *χ*_+_, $R_{2,0}^{*}$) and linear parameters (*C*_+_, *C*_−_, *C*_0_). If only the linear parameters were to be estimated, the problem would have been perfectly convex, and a least square objective function would have sufficed. However, if the least square option is used as the objective function, the optimization will be largely dominated by the magnitude effect as tissue-susceptibility induces relatively small phase shift. On the other hand, if only nonlinear parameters were to be estimated, taking a logarithm of the modeled signal will linearize the model and ensure phase information weighs significantly in the objective function. With these considerations, we divide the optimization problem of Eq. (3) into three sub-problems as follows,

(5.1) $$\underset{C_{+},C_{-},C_{0}}{\text{min}}{\left\|S\left(C_{+},C_{-},C_{0};t,\chi_{+},\chi_{-},R_{2,0}^{*}\right)-y(t)\right\|}_{2}\;\;s\text{.}t\text{. : }C_{+}+C_{-}+C_{0}=1\;0<C_{+,-,0}<1$$

(5.2) $$\underset{R_{2,0}^{*}}{\text{min}}{\left\|\text{log}\left(S\left(R_{2,0}^{*};t,C_{+},C_{-},C_{0},\chi_{+},\chi_{-}\right)\right)-\text{log}(y(t))\right\|}_{2}\;\;s\text{.}t\text{. : }R_{2,0}^{*}>0$$

(5.3) $$\underset{\chi_{+},\chi_{-}}{\text{min}}{\left\|\text{log}\left(S\left(\chi_{+},\chi_{-},;t,C_{+},C_{-},C_{0},R_{2,0}^{*}\right)\right)-\text{log}(y(t))\right\|}_{2}\;\;s\text{.}t\text{. : }0.5>\left|\chi_{+}\right|,\left|\chi_{-}\right|>0$$

The upper bound constraint of |*χ*_+_|, |*χ*_−_| is roughly calculated using TE = 25 ms and B_0_ = 3T to ensure the phase does not exceed 2π. The estimation for $R_{2,0}^{*}$ has been singled out because this parameter is a linear parameter after taking the logarithm (Eq. (5.2)). The modification of taking the logarithm in Eq. (5.2) and Eq. (5.3) will not change the optimality since logarithm is monotonically increasing for variables > 0. The logarithmic operation is not performed while solving for linear parameters (Eq. (5.1)) to preserve the simplicity of the constrained linear program. We solve these three sub-problems in an alternating and iterative fashion (Fig. 1B). With the computational cost in mind, we find that alternating 10 iterations among 3 steps is sufficient to achieve the optimality (Fig. S1).

### 2.3. Paramagnetic susceptibility Component (PSC) and Diamagnetic susceptibility Component (DSC)

The model yields six estimated parameters. While we can simply use *C*_+_*χ*_+_ and *C*_−_*χ*_−_ to quantify the sub-voxel paramagnetic susceptibility and diamagnetic susceptibility respectively, such an approach does not fully account for the complex tissue environment. Instead, we define a Paramagnetic Component Susceptibility (PCS) and a Diamagnetic Component Susceptibility (DCS) computed based on the signal model as follows,

(6) $$\text{PCS}=\frac{\sum_{t}∡\left(C_{+}e^{-\left(a\chi_{+}+R_{2,0}^{*}+i\;\frac{2}{3}\;\chi_{+}\gamma B0\right)t}+\left(C_{0}+C_{-}\right)e^{-R_{2,0}^{*}t}\right)}{\frac{2}{3}\gamma B_{0}\sum t}$$

(7) $$\text{DCS}=\frac{\sum_{t}∡\left(C_{-}e^{-\left(-a\chi_{-}+R_{2,0}^{*}+i\;\frac{2}{3}\;\chi_{-}\gamma B0\right)t}+\left(C_{0}+C_{+}\right)e^{-R_{2,0}^{*}t}\right)}{\frac{2}{3}\gamma B_{0}\sum t}$$

The PCS or DCS represents the situation of a voxel where only paramagnetic or diamagnetic component exists along with neutral medium. The quantity of each is nonlinear with respect to the bulk susceptibility.

Likewise, the composite susceptibility map is then defined as

(8) $$\text{Composite susceptibility }=\frac{\sum_{t}∡\left(\left(C_{+}e^{-\left(a\chi_{+}+i\;\frac{2}{3}\;\chi_{+}\gamma B0\right)t}+C_{-}e^{-\left(-a\chi_{-}+i\;\frac{2}{3}\;\chi_{-}\gamma B0\right)t}+C_{0}\right)e^{-R_{2,0}^{*}t}\right)}{\frac{2}{3}\gamma B_{0}\sum t}$$

Since χ_±_ are defined as the volume susceptibilities of the sources, rather than bulk susceptibility, the herein defined PCS or DCS can be viewed as the bulk susceptibility with only one susceptibility sources and the reference susceptibility medium. PCS, DCS and the composite susceptibility are the effective QSM and are the comparable measures with conventional QSM. Later, we show that PCS and DCS can be reliably estimated and are less sensitive to the choice of “a” in Eq. (1).

## 3. Material and methods

### 3.1. Implementation of the proposed algorithm

The algorithm and all the computing procedures are implemented in MATLAB 9.7 (The Math Works, Inc. MATLAB. Version 2019b) with Parallel Computing Toolbox and Optimization Toolbox running on a MacBook Pro with 2.8 GHz Intel core i7 processor and with 16 GB memory. Particularly, Eqs. (5.1) and (5.2) are solved by “interior-point” methods with “@lsqlin” function and Eq. (5.3) is solved through “trust-region-reflective” method with “@lsqnonlin” using manually calculated Jacobian sparse pattern to accelerate. The algorithm will be released under STI Suite (https://people.eecs.berkeley.edu/~chunlei.liu/software.html).

### 3.2. Simulation

**Fig. 2.**
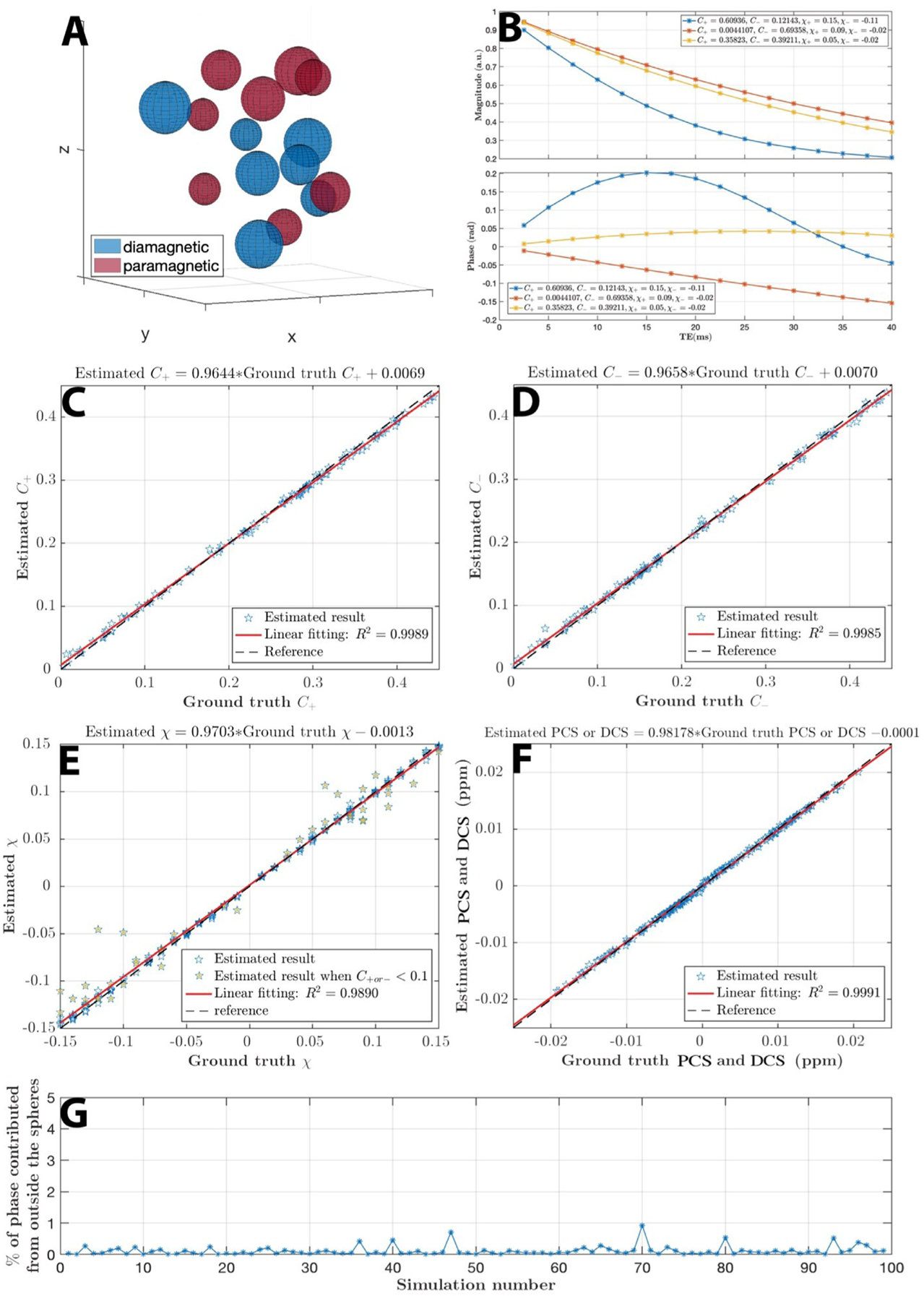
Numerical simulation. (A) Spheres assigned either positive or negative susceptibility with various radius are drawn randomly in a voxel space. (B) Illustration of magnitude and phase signal progression for 3 simulated voxels with different combinations of assigned parameters.(C ~ E) Estimated parameters versus the ground truth are shown respectively. (E) The simulation results mostly are in a good agreement with ground truths. Results of χ that deviate the most from the ground truth are the cases when the concentration is low (*C*_+*or*−_ < 0.1). (F) The composite paramagnetic component susceptibility (PCS) and diamagnetic component susceptibility (DCS) shows good agreement with the ground truth. (G) The magnetic field perturbation outside the spheres has negligible contribution to the total phase of the voxel as shown over 100 random simulations, which confirms the validity of the voxel field approximation.

The analytical forward field simulation was performed for 100 voxels independently with each of them consisting of 128^3^ sub-voxels with TE_1_/spacing/TE_16_ = 2.5/2.5/40 ms, TR = 42 ms, B_0_ = 3T. Simulations are running with MATLAB 9.7 (The Math Works, Inc. MATLAB. Version 2019b) on an Ubuntu 18.04.5 64-bit platform and 48 CPUs of Intel(R) Xeon(R) Silver 4116 CPU with 2.10 GHz, 502 GB memory. For each simulation (i.e., each of the 100 voxels), spheres with varying radius and pre-assigned susceptibility (χ = 0.01 ~ 0.15 ppm) representing either diamagnetic or paramagnetic component are generated randomly within this 128^3^ cubic (Fig. 2A). The radius of these spheres was forced to be greater than 6 sub-voxels to reduce numerical error of digitizing a sphere. Histology study of common pathological plaques with iron and protein aggregation are around the size of tens to hundreds of microns(Aguzzi and O’Connor, 2010; Meadowcroft et al., 2009; Verwilst et al., 2018); the randomized choices of susceptibility source’s radius are to imitate such sub-voxel situations. Then a B_0_ field aligning with the z direction of the voxel is applied. Induced forward field perturbation was calculated in an analytical fashion for each sub-voxel using the superposition rule of the fields produced by the spheres. The GRE signal of each sub-voxel is generated by a single component exponential function with its magnitude being an exponential decay $\left(R_{2}^{*}=20\;\text{Hz}\right)$ and its phase being proportional to the corresponding field perturbation at that sub-voxel. The signals from each of the 128^3^ sub-voxels are summed together assuming equal proton density to form the total complex signal of the simulated voxel.

### 3.3. Agarose gel phantom fabrication

Agarose (Fisher Scientific) was mixed with water at 1% w./w. (with *T*_2_ is approximately 60 ms according to (Yoshimura et al., 2003)) to achieve a typical *T*_2_ of biological tissue. The agarose water mixture was heated up with a microwave oven until it is forming homogeneous clear liquid. The agarose gel solution is poured into a 1L cylindrical Nalgene jar (112 mm diameter and 151 mm height) made from Polypropylene with seven cylindric place holders. After the agarose solution solidified, 20 mL holes (17.5 mm diameter) were made with smooth surface so that signal gaps and air bubbles can be avoided. These holes are then filled with agarose gel mixed with different concentrations of susceptibility sources to form a direct contact with the outer embedding agarose gel. We use CaCO_3_ (Sigma-Aldrich) as the diamagnetic species and Fe_2_O_3_ (Sigma-Aldrich) as the paramagnetic species. Two calibration phantoms were fabricated with gradient concentrations of Fe_2_O_3_ (or CaCO_3_) to achieve estimated susceptibility ranging from 0 ~ 0.15 ppm (or −0.1~0 ppm for CaCO3) to mimic typical biologic tissue volume susceptibilities. One susceptibility mixture phantom was made to validate the proposed model and algorithm. The mixture ratio of each cylinder is indicated in Fig. 4K. The detailed parameters are listed in the supporting tables (Table S1, S2).

### 3.4. Phantom and human MRI acquisition

The phantoms were scanned on a GE MR750w 3T scanner (GE Healthcare, Milwaukee, WI, USA). Each phantom was placed in the scanner with its long cylindrical axis aligned with the B0 field of the scanner and was scanned axially with a three-dimensional multi-echo gradient echo (GRE) sequence of the following scan parameters: TE_1_/spacing/TE_16_ = 1.47/1.63/25.9 ms, TR = 30.1 ms, bandwidth = 62.5 kHz, matrix size = 192 × 192 × 128, and a native spatial resolution of 1.0 × 1.0 × 1.0 mm^3^.

Raw complex images of ten PD patients and ten healthy subjects from a previous study (He et al., 2015) were used in this paper. Imaging parameters for the multi-echo GRE sequence prescribed on the axial plane were as follows: TE_1_/spacing/TE_16_ = 2.7/2.9/46.2 ms, TR = 59.3 ms, bandwidth = 62.5 kHz and a spatial resolution of 0.86 × 0.86 × 1.0 mm^3^.

### 3.5. Ex vivo brainstem imaging

Following Curie’s Law, over a certain range of temperature and field strength, paramagnetic susceptibility is approximately inversely proportional to temperature,

(9) $$\chi (T\to \infty )=\frac{C}{T},$$

where C is the material’s specific Curie constant. We use this relationship to validate the resulting PCS. Specifically, the PCS is expected to be temperature dependent as paramagnetic susceptibility in brain tissue is predominately caused by iron while the DCS should remain stable as temperature changes. Such an effect is visible through QSM according to a previous study (Birkl et al., 2015).

Two human brainstems were fixed in non-buffered 4% formalin for over 12 months, individually, washed out in distilled water and placed into a proton-free fluid called Fomblin (a chemically inert perfluoropolyether fluorocarbon) prior to MRI scanning. Fomblin produces no MRI signal and has a similar magnetic susceptibility to tissue (Shatil et al., 2016). Sample tube was heated up using a hot water bath for 3 hours at 40°C just before MRI scans. MRI acquisition was performed using a 7T Magnetom (SIEMENS, Erlangen, Germany) with a gradient amplitude of 70mT/m and a slew rate of 200 T/m/s, using an in-house built solenoid coil with 28 mm of inner diameter, 34 mm outer diameter and 130 mm length. Just before placing the sample into the scanner, temperature was measured using a digital infrared thermometer.

QSM acquisition was repeated within 11 hours, while the brainstem was allowed to cool down naturally until reaching thermal equilibrium at room temperature (20°C). One sample was scanned with five echoes of TE_1_/deltaTE/TE_5_ = 4/3/16 ms, TR = 32 ms, and the other with 16 echoes of TE_1_/deltaTE/TE_16_ = 4/3/49 ms, TR = 64 ms. Acquisition time for each QSM sequence was 17 min 20 sec and 11 min 33 sec, respectively. For the 16-echo acquisition, due to the low SNR of images at later echoes, only the first 12 echoes were adopted for further analysis.

Just before every new GRE sequence a single-shot water-unsuppressed spectrum was acquired, using a semiLASER sequence (Deelchand et al., 2019; Scheenen et al., 2008; Slotboom and Bovée, 1995), with TE/TM/TR = 7/26/9000ms. The size of the voxel was 30 × 20 × 20mm^3^ including most of the brainstem. The acquired spectrum allowed to measure water chemical shift as a function of time, which was used to calibrate sample temperature (Fig. S4). For the 16-echo data, due to a sequence error, the spectrum information was not properly saved. The initial temperature before placing the brainstem into the scanner was recorded to be 36°C and the end temperature was 21°C. Fig. S5 and S6 were drawn assuming the temperature change during natural cool down is similar as that in the 5-echo data.

### 3.6. Image preparation and QSM reconstruction

QSM reconstruction was performed with functions in STI Suite V3.0 (https://people.eecs.berkeley.edu/~chunlei.liu/software.html). The GRE magnitude images of the first echo was used to mask and extract the brain tissue using the brain extraction tool (BET) in FSL(Smith et al., 2004). The raw phase was unwrapped using a Laplacian-based phase unwrapping method (Li et al., 2011; Schofield and Zhu, 2003).The background phase was removed with the V-SHARP method(Li et al., 2018; Wu et al., 2012b). Lastly, STAR-QSM (Wei et al., 2015) was performed to compute the susceptibility maps for each echo. For the in vivo scans, susceptibility values were referenced to the mean susceptibility of the whole brain as it has previously been shown that no obvious systematic bias is observed between analysis with and without referencing to CSF (Li et al., 2014). For the ex vivo scans, due to the lack of a common reference for QSM across the temperatures, the resulting ex vivo temperature dependent QSM maps are re-referenced to the region where $R_{2}^{*}$ value has lower than 2 Hz standard deviation over time.

## 4. Results

### 4.1. Forward numerical simulation

The analytical forward simulation is performed to justify the assumed model as well as to measure the ability of the proposed solver for the noiseless case. The forward field and signal simulation requires ~4 hours per voxel. With 24 CPUs parallel computing, simulating 100 voxels can be completed in two days. Simulation shows that the magnitude profile of the mixture model remains largely exponential as a function of TE, while the phase develops a nonlinear TE dependency (Fig. 2B), consistent with previous reports of TE-dependent QSM in the brain (Cronin et al., 2017; Sood et al., 2016). These results demonstrate that 1) it is necessary to use TE-dependent QSM as the input, and 2) phase information needs to play a significant role in the objective function to alleviate the difficulty of separating the summation of exponentials.

Parameter estimation results versus ground truths are shown in Fig. 2C–F. In general, estimations for linear parameters (*C*_+_, *C*_−_, *C*_0_) show less deviations from ground truths than those of the nonlinear parameters (*χ*_+_, *χ*_−_). Nevertheless, the composite paramagnetic component susceptibility (PCS) and diamagnetic component susceptibility (DCS) agree with the ground truth. The forward field simulation also confirms that the assumption of “total field perturbation contributed from outside sphere is nearly zero” is valid (Fig. 2G).

### 4.2. Gel phantom imaging

**Fig. 3.**
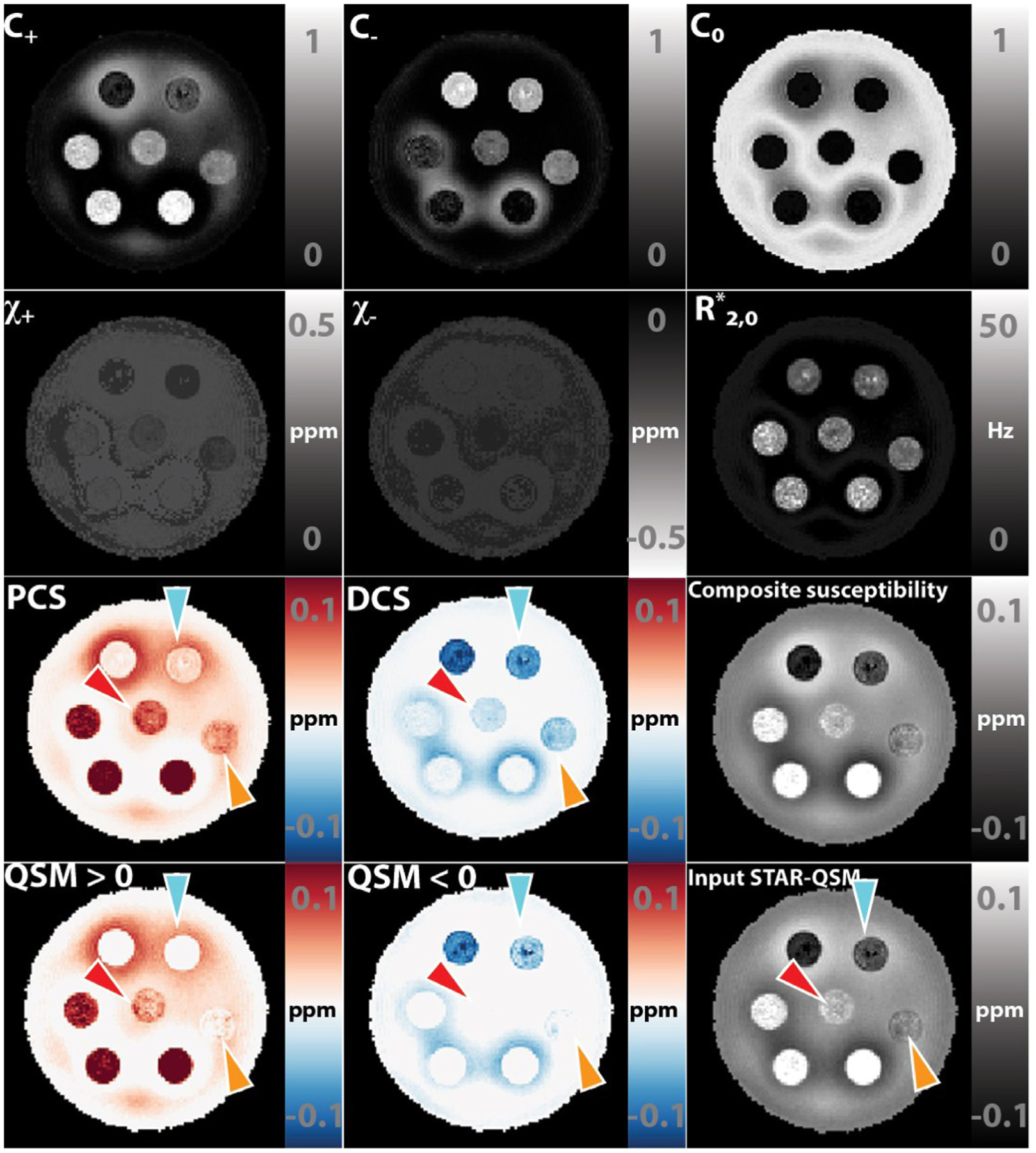
DECOMPOSE-QSM results of a susceptibility-mixture phantom showing the parameters and composite susceptibility maps in comparison with thresholding original QSM. Note that the subplots that relate to the diamagnetic component are displayed with inverted dynamic range to have a better visual contrast. The composition of each tube is shown in Fig. 4K. Arrows point at the regions of interest that show visually significant improvement in the contrast of the mixture.

Fig. 3 illustrates the maps of each estimated parameters and the composite maps. The halo-looking artifact is the streaking artifacts viewed in the axial slice from QSM inversion. The inversion algorithm, STAR-QSM, is optimized for in vivo susceptibility calculation, where susceptibility map of bio-tissue should not have sharp edges. The existence of such artifact is due to the sharp transition of susceptibility at the boundary of ROIs. The *C*_0_ map successfully captured the region where the material is purely agarose gel without any susceptibility species (referenced to water). The PCS and DCS maps verify that DECOMPOSE-QSM is able to reveal the mixing situation of each cylinder while threshold QSM can not reveal such information. This is especially striking in cylinders where the paramagnetic and diamagnetic components’ contributions cancel out leading to nearly zero value in the original QSM.

**Fig. 4.**
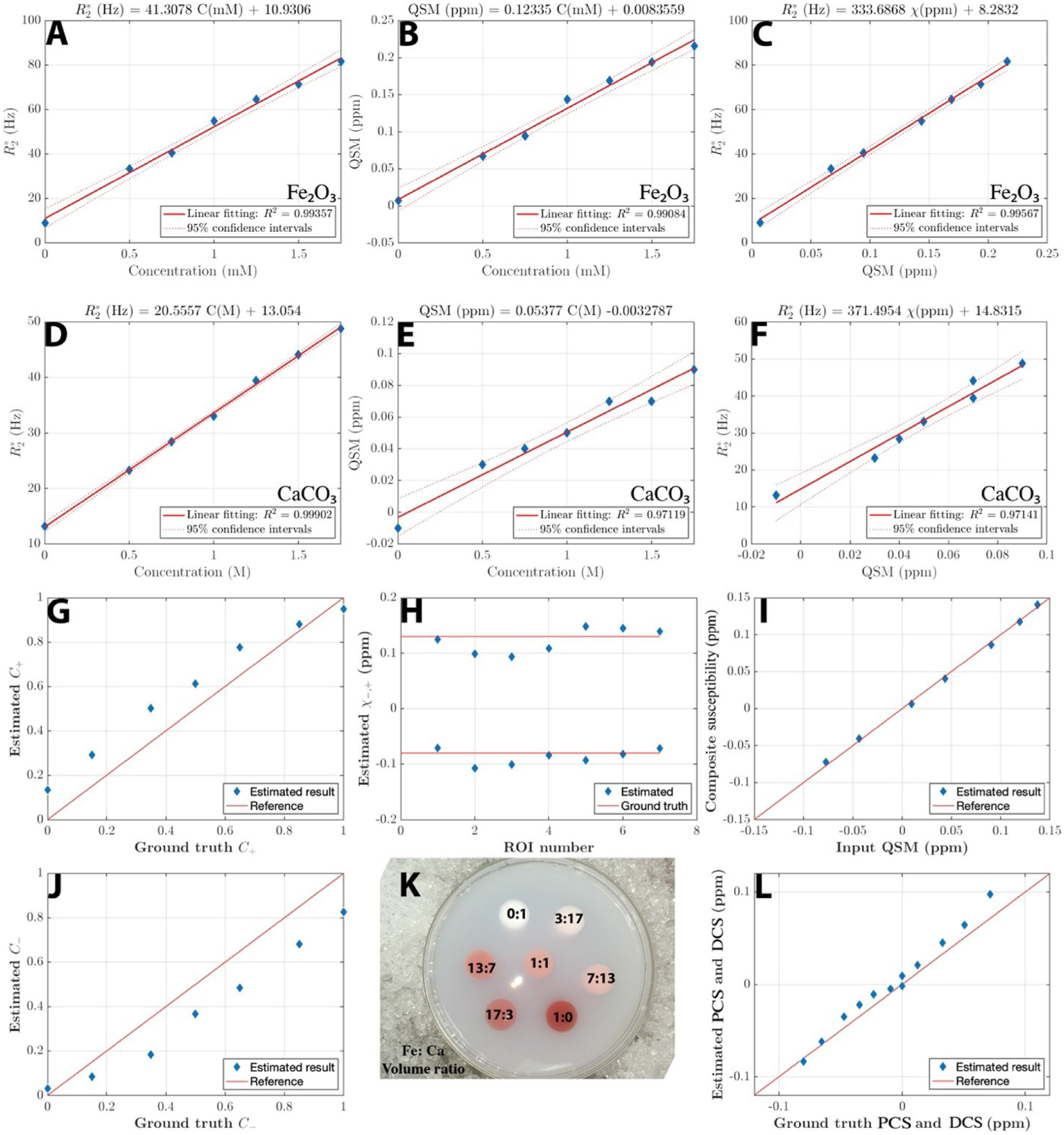
Phantom experiments. (A,B,D,E) Linear regression of ${\text{R}}_{2}^{*}$ and QSM versus concentrations of each species from each calibration phantom. (C~F) Linear regression of ${\text{R}}_{2}^{*}$ versus QSM for each calibration phantom. The linear slopes match the derivation from static regime theory. (G~L) Parameters estimated with DECOMPOSE-QSM versus ground truths. Red solid lines are the reference lines when ground truth equals estimation. The composite susceptibility of each ROI in (I) agrees with input. PCS and DCS in (L) shows good alignment with ground truth. (K) is a top view photo of the susceptibility mixture phantom.

The two calibration phantoms showed a high linearity of $R_{2}^{*}$ vs QSM (Fig. 4A–F). The linear slope of ${\text{R}}_{2,+,-}^{*}\sim \chi_{+,-}$ is estimated to be 334 Hz/ppm for the Fe_2_O_3_ phantom and 371 Hz/ppm for the CaCO_3_ phantom. These values agree with the theoretical calculation of 323.5 Hz/ppm.

As shown in Fig. 4G–L, the DECOMPOSE-QSM calculation is able to separate the paramagnetic and diamagnetic components. Despite some inaccuracy, the estimated *C*_+_, *C*_−_, *χ*_+_, *χ*_−_ values largely lie close to the reference red solid line that indicates where a perfect estimation would fall onto. On the other hand, the paramagnetic component susceptibility (PCS) and diamagnetic component susceptibility (DCS) estimations are highly accurate. The composite susceptibilities align with the input mean QSM (Fig. 4I,). Detailed numbers are included in Table S1 and S2.

A Previous study has investigated the potential phase temporal artifact caused by Laplacian based unwrapping and filtering (Cronin et al., 2017). To validate that the nonlinear phase evolution we observed is not a confounding result from Laplacian based phase unwrapping (Li et al., 2018; Wu et al., 2012b)), temporal based phase unwrapping (Liu et al., 2009) was performed for each echo (Fig. S2). Temporal unwrapping retained significant spatial phase wraps when the field inhomogeneity is too large for the echo spacing of this acquisition (ΔTE = 1.63 ms) or when SNR is low (Fig. S2). Despite that, it is shown that at the earlier echoes, at regions when temporal-based phase unwrapping is successful, nonlinearity of the phase progression was still observed and was similar to that of Laplacian based unwrapping.

### 4.3. Temperature variant ex vivo brainstem imaging

**Fig. 5.**
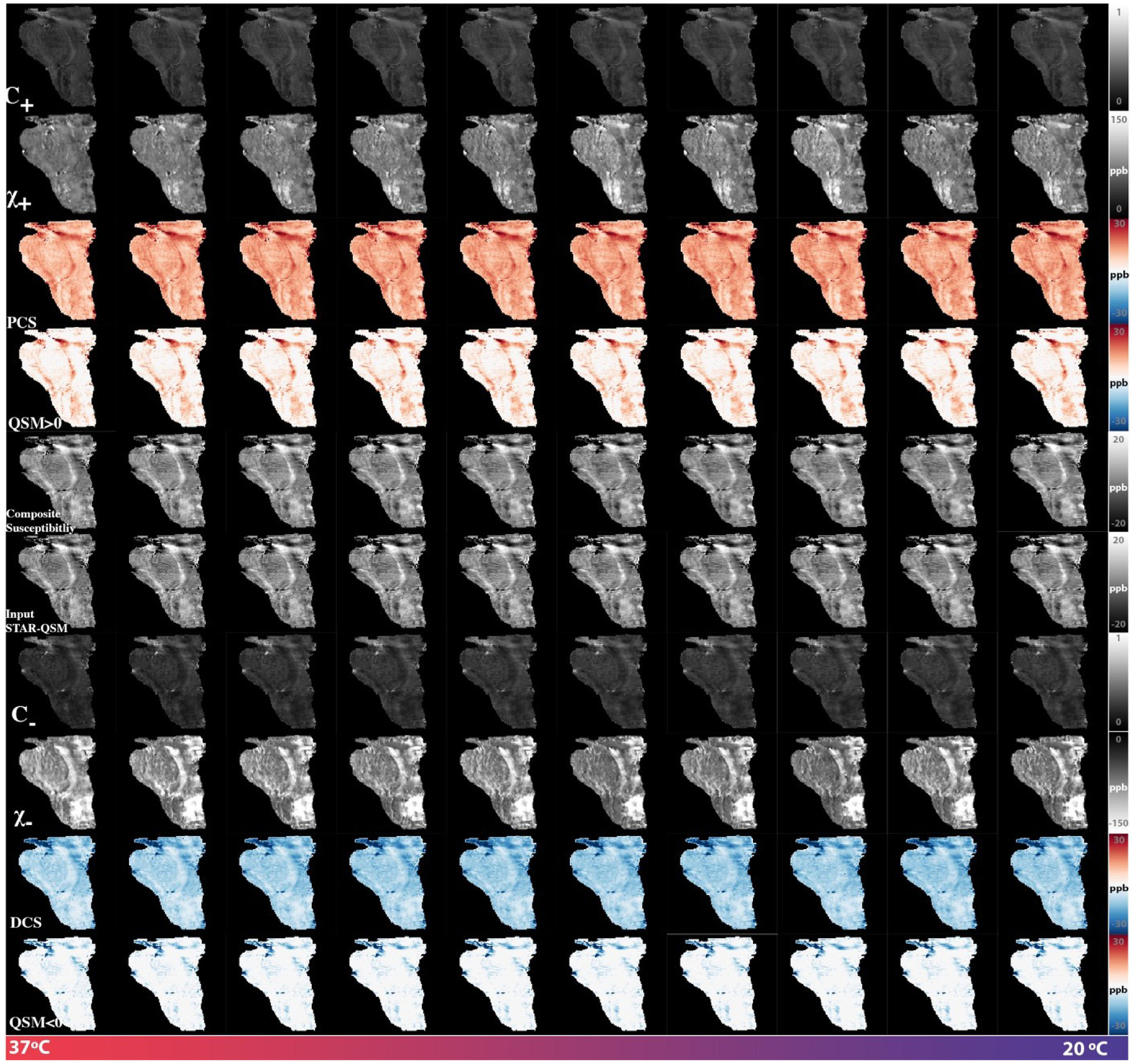
DECOMPOSE-QSM parameter maps of a brain stem specimen as a function of temperature. While DCS maps remain mostly stable, PCS maps show an increasing trend especially for the first five scans where temperature was changing the most drastically. The subplots relating to χ_−_ and DCS are displayed with inverted dynamic range to have a better visual contrast.

**Fig. 6.**
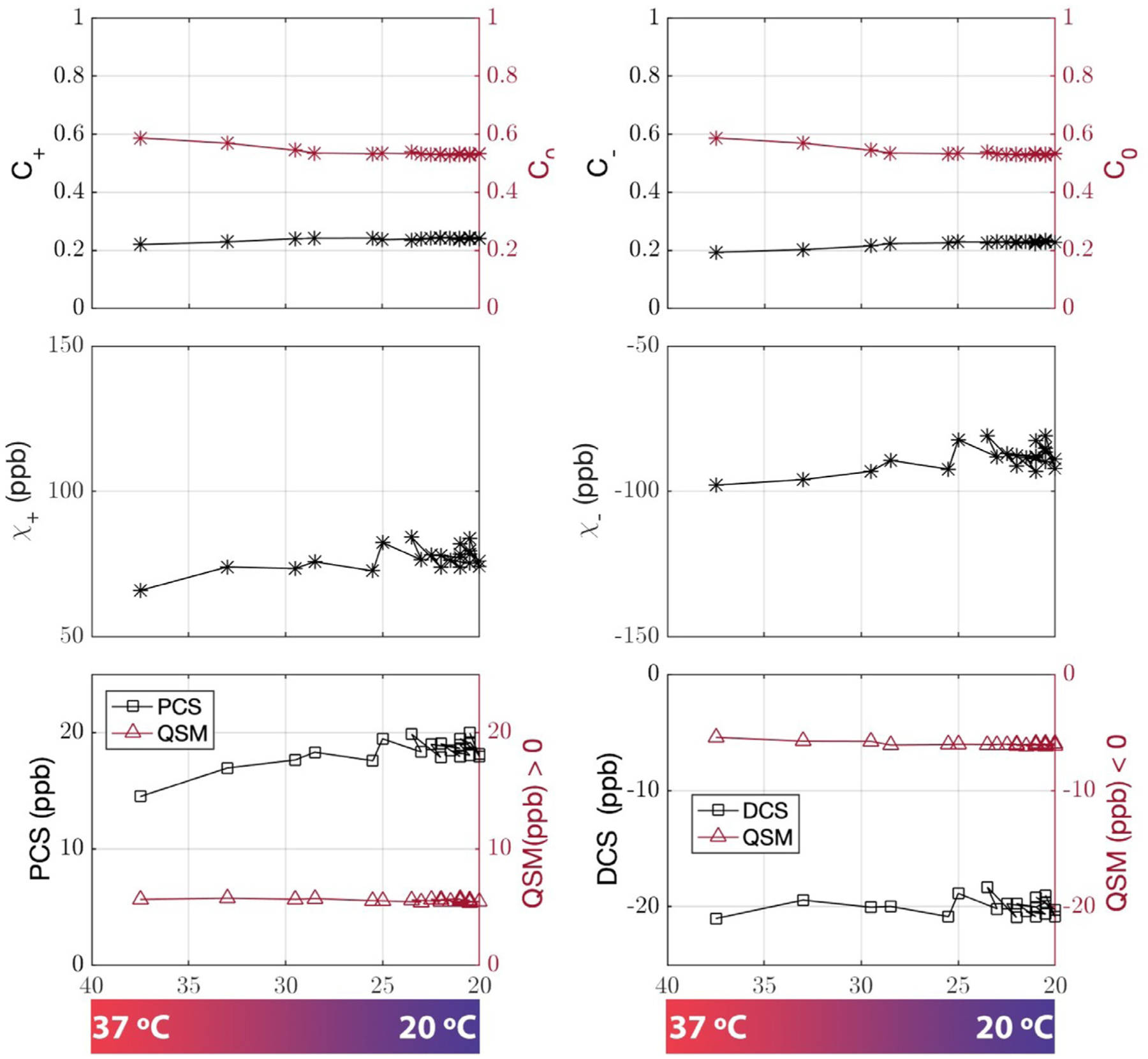
DECOMPOSE-QSM results of a brain stem specimen as a function of temperature. GRE data were acquired with five echoes. Temperature ranges from 37 °C to 20 °C. The mean value of each parameter of a representative slice is displayed vs. temperature changes. The paramagnetic component susceptibility (PCS) shows an increasing trend that’s more prominent than the corresponding threshold QSM.

**Fig. 7.**
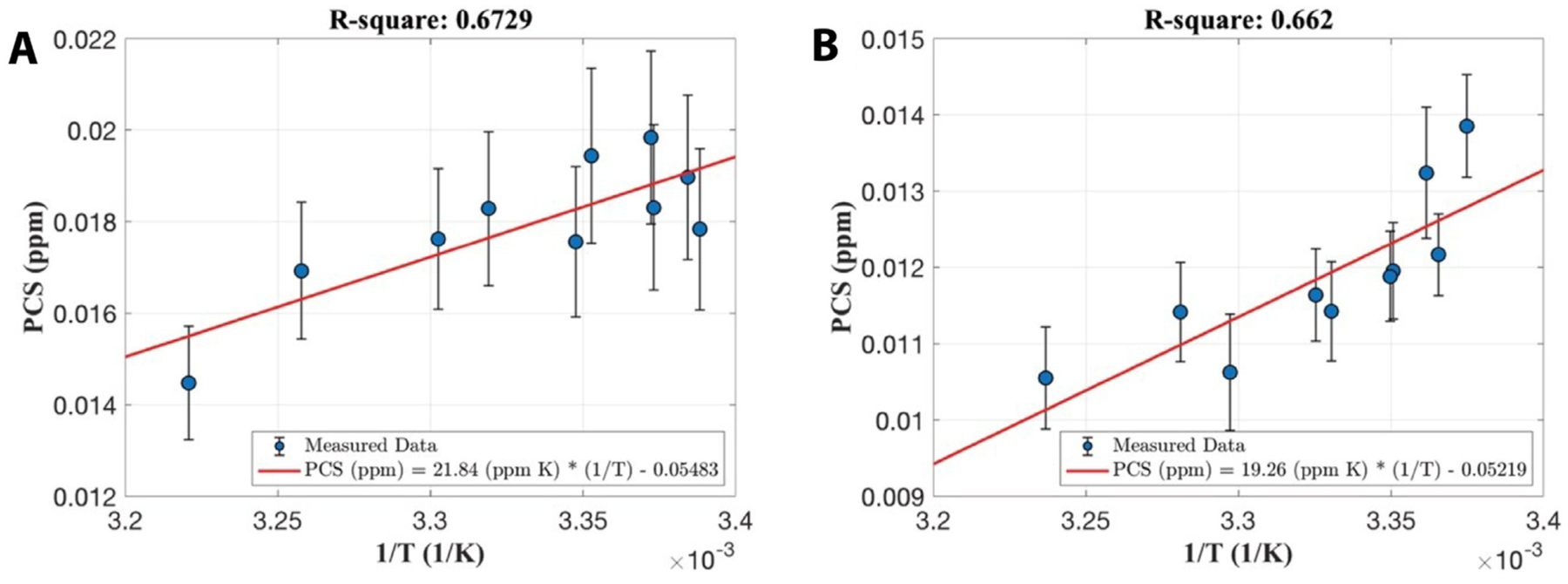
Linear correlations of paramagnetic component susceptibility with the inverse of temperature from data with five-echo data (A) and twelve echoes (B). Each error bar is the standard deviation of PCS of the same sagittal slice of each temperature acquisition.

DECOMPOSE-QSM was also validated using the fact that paramagnetic susceptibility is temperature dependent. Temperature of the brainstem was estimated using water proton chemical shift (Fig. S4). The DECOMPOSE results of each set of data are presented as line graphs and corresponding parameter maps. Detailed parameter maps of one sagittal slice of each specimen are shown in Fig. 5 and Fig. S5. The resulting PCS showed more visible increases than threshold QSM maps as the temperature decreases before reaching the thermal equilibrium of room temperature. The DCS showed minimal changes across the scans. With both 5 and 12 echoes, the increases in the resulting PCS are visually noticeable (the increase of red color from left to right in the row of PCS) in the maps as the temperature decreases. This trend is also shown as line plots in Fig. 6 and S6. It appears that the 12-echo data result in more stable DCS maps across the temperatures. We further estimated the Curie constant for the brainstem tissue by linear fitting of PCS with the reciprocal of temperature (Fig. 7). The Curie constant is estimated to be 21.84 ppm K with the 5-echo data. If the temperature change during natural cool down is similar for both experiments, we estimated the Curie constant from data with 12 echoes to be 19.26 ppm K. Curie constant of brain tissue was previously estimated at around 2 ppm emu K/g/Oe (Birkl et al., 2015), which is 25 ppm K if the density of brain tissue is approximate 1g/cm^3^. Our estimation is therefore comparable with the reported value of brain tissue.

### 4.4. In vivo human brain imaging

**Fig. 8.**
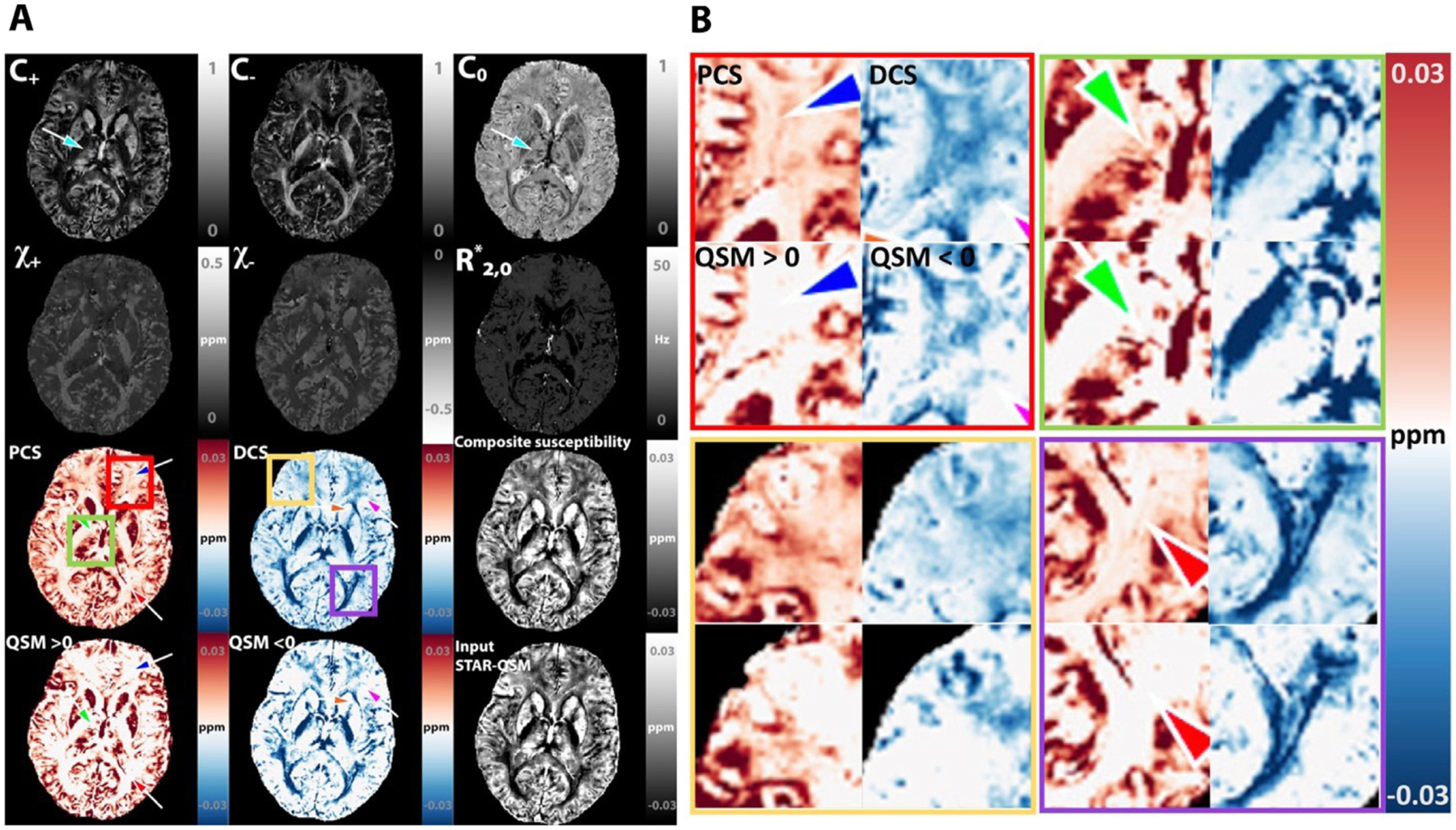
DECOMPOSE-QSM of a healthy adult study participant. (A) Individual parameter maps of DECOMPOSE results. First row: signal fraction maps show high fraction of paramagnetic susceptibility in gray matter, high fraction of diamagnetic susceptibility in white matter and high fraction of neutral component in the ventricles. The C_0_ map particularly reveals clear delineation of the thalamic subnuclei (arrow). Third and fourth row: The paramagnetic component susceptibility (PCS) and diamagnetic component susceptibility (DCS) show the existence of sub-voxel mixture of paramagnetic and diamagnetic components in both gray and white matter (arrows), which is not revealed in threshold QSM. The composite susceptibility is comparable to the input STAR-QSM. The subplots relate to the χ_−_ and DCS are displayed with inverted dynamic range to have a better visual contrast. (B) zoom-in view of four regions.

Fig. 8 shows a representative case of the DECOMPOSE being applied to a healthy subject’s brain. Additional illustrations of another healthy subject are shown in Fig. S7. The calculation time of one axial slice is ~250 seconds on a MacBook Pro with 2.8 GHz Intel core i7 processor and with 16 GB memory. For 16 echoes of the signal, parameter estimation converges at majority region of brain tissue with squared residual less than 0.1. The fitted complex signals for four representative voxels illustrate the general goodness of fitting (Fig. S8). The PCS and DCS show a good agreement with the known distribution of paramagnetic and diamagnetic species in human cerebral region (Fig. 8 and S7). The DCS shows more complete white matter regions than simply taking a threshold of QSM. The *C*_0_ map showed highest values at the atrium of the lateral ventricles and the anterior horns of the ventricles. These high values of *C*_0_ correspond to the high concentration of cerebrospinal fluid (CSF) which has high free water fraction and low susceptibility in those regions. Interestingly, the *C*_+_, *C*_−_, *C*_0_ map reveal distinctive structural boundaries between subfields of thalamus, reflecting a variation of free water concentration among the various brain structures. The composite susceptibility shows minimal difference from the input QSM. This confirms that DECOMPOSE-QSM preserves the total volume susceptibility value.

Further, we compare DECOMPOSE results at frontal cortex regions with the histology data reported in (Stüber et al., 2014). As shown in Fig. S9, comparing to the threshold QSM, PCS reveals the iron content in cortical white mater and DCS map matches the pattern of the myelin content, visually more consistent with histology.

**Fig. 9.**
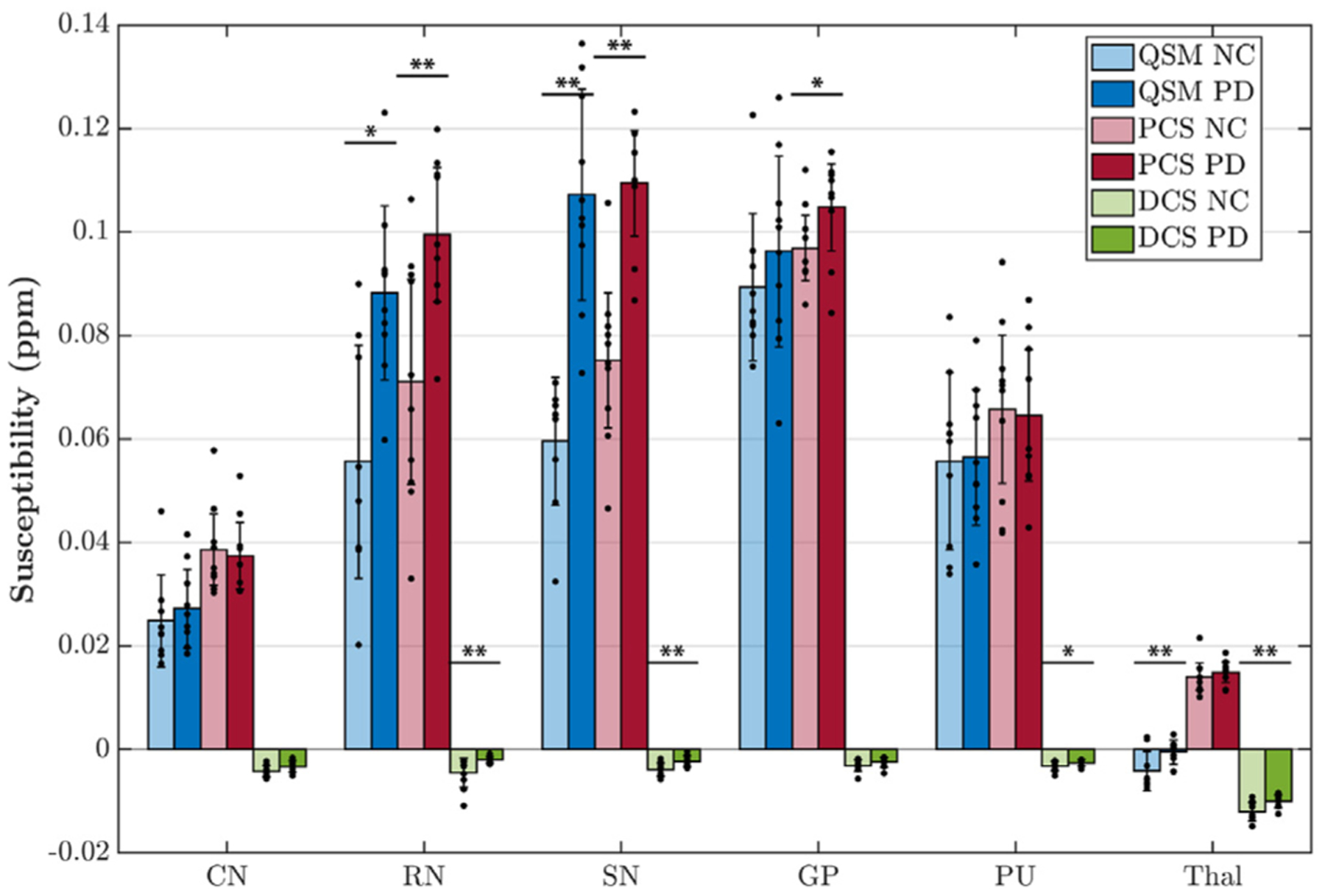
Region-of-interest (ROI) analysis of PD patients vs. controls (n = 10) for QSM, paramagnetic component susceptibility (PCS) and diamagnetic component susceptibility (DCS). Susceptibility values of each contrast and each ROI are shown as bars with standard deviation presented as error bar. Data points of each ROI from each subject is shown as black dots overlayed on the bar graph. Symbols of “*” indicate significant difference: *p < 0.05, **p < 0.01. CN: caudate nucleus; RN: red nucleus; SN: substantia nigra; GP: global pallidus; PU: putamen; Thal: thalamus.

One-tailed two-sample t-test shows that DECOMPOSE-QSM can detect known susceptibility differences between PD patients and healthy controls in various brain regions. Nuclei in basal ganglia and substantia nigra are known to be involved in PD progression (DeLong and Wichmann, 2015; Obeso et al., 2000). Iron alternations in regions of caudate nucleus (CN), red nucleus (RN), substantia nigra (SN), global pallidus (GP), putamen (PU) and Thalamus (Thal) are often being investigated to improve the understanding of PD pathology (Barbosa et al., 2015; Guan et al., 2019; He et al., 2015; Langkammer et al., 2016). The regions with statistically significant difference between PD patients and controls in QSM also show significant difference in PCS (or DCS for thalamus, Fig. 9). In the thalamus region, the mean QSM of the region has a negative value. After decomposing, the DCS showed significant a difference, but PCS did not. Interestingly, DCS of RN, SN and PU also showed significant differences. The detailed values are displayed in Table S3.

## 5. Discussion

QSM is an increasingly used MRI technique for quantifying tissue magnetic susceptibility. However, biologic tissues are generally complex and MRI resolution is limited. As a result, QSM does not characterize the sub-voxel distribution of magnetic susceptibility. Here, we propose and develop DECOMPOSE-QSM to separate the diamagnetic and paramagnetic susceptibility components within a voxel. The method is theorized based on GRE signal behaviors in compartmentalized tissue microstructures and validated with numerical simulation, phantom experiments, ex vivo and in vivo brain imaging experiments.

### 5.1. The estimations of *C*_+_*, C*_−_*, C*_0_, *χ*_+_, *χ*_−_, $R_{2,0}^{*}$

In general, the estimations of *C*_+_, *C*_−_, *C*_0_ show a high accuracy (in the numerical simulation, Fig. 2) and yield reasonable concentration maps (Fig.s 3, 5, 8 S5 and S7). Particularly, the *C*_0_ map in the phantom experiment (Fig. 3) well captures the pure gel portion of the susceptibility mixture phantom. The *C*_0_ map reveals a high reference medium concentration in the ventricle CSF (nearly 1 in value); it also shows anatomical meaningful subfields of the thalamus and putamen region (Fig. 8 and S7). These results suggest that a high *C*_0_ map may indicate a low level of cell density or high free water concentration. Further studies are needed to compare these results from similar parameters estimated with other MRI methods, such as, diffusion based NODDI (Gong et al., 2020; Yi et al., 2019; Zhang et al., 2012)

The estimations of *χ*_+_ and *χ*_−_ are similarly highly accurate when *C*_+ *or* −_ > 0.1, however the accuracy decreases when *C*_+_ or *C*_−_ has relatively small values (*C*_+ *or* −_ < 0.1) (Fig. 2E). For example, in the internal capsule (Fig. 8 and S7), *C*_+_ has a relatively lower value, and *χ*_+_ is unexpectedly higher than that of the global pallidus region. Although one may speculate that this difference suggests a different paramagnetic molecular species in the internal capsule that is more paramagnetic than that of the global pallidus, it is more likely that this difference is an estimation inaccuracy. Another example can be found in the parameter maps of the phantom experiments (Fig. 3). In the cylinders with high iron concentration, *χ*_+_ is estimated accurately; while in cylinders with low iron concentrations, *χ*_+_ is underestimated compared to the true value. Similar cases occur in the *χ*_−_ maps. This inaccuracy can be explained as follows. When concentration level, *C*, is low, a slight alternation in the value of *χ* will not lead to a significant change in the objective function evaluation due to the multiplicative relationship. The solver captures the nonlinear signal progression to estimate parameters rather than linear superposition of positive and negative species. However, at low concentration, the nonlinearity is less significant. Therefore, the inaccuracy of nonlinear parameters appears.

The above reasoning also explains that despite the slight inaccuracy of nonlinear parameters’ estimations, the composite susceptibility maps, PCS and DCS are still highly accurate to reveal the sub-voxel susceptibility mixing situation (Fig. 2F). PCS and DCS are considered to be effective QSM. For example, PCS is the estimated bulk susceptibility as if the negative susceptibility sources within the voxel are replaced with the reference susceptibility medium. While one can always use each of the estimated parameters for further analysis (Fig. S11), obtaining accurate values of χ is fundamentally challenged due to the high nonlinearity of Eq. 2. On the other hand, the effective QSM, PCS and DCS are not only insensitive to the estimation error of χ but also provide comparable values with QSM.

In our model, the $R_{2,0}^{*}$ parameter is the apparent transverse relaxation rate of the reference susceptibility component. $R_{2,0}^{*}$ includes contributions from R_2_ and $R_{2}^{\prime }$ as the protons experience in the background medium (i.e., the “reference susceptibility” source). Both diamagnetic and paramagnetic susceptibility sources contribute to the $R_{2}^{\prime }$ decay of the medium even though their phase contributions average to be zero within the medium (Fig. 2G). Further, calcium and iron also affect water R_2_ (Gamsu et al., 1987; Schenck, 1995; Vymazal et al., 1996).

### 5.2. Choices of echo times

The DECOMPOSE method is based on multi-echo 3D GRE data. Generally, the more echoes are available, the more beneficial it is to the algorithm as the model relies on the temporal behavior of the signal progression. Here we show that, in practice, with as few as five echoes, the algorithm is still able to separate paramagnetic and diamagnetic susceptibility. In general, the echo times should be well spaced for a good balance between SNR and sufficient phase variation accumulation. While short TE offers better SNR, it captures very limited phase variation. On the other hand, at longer TE, the SNR is too poor to provide useful signal. The range of TE should generally cover the corresponding T_2_* of the tissue (Wu et al., 2012).

### 5.3. Linear coefficient a of *R*_2_* and single source susceptibility *χ*

The parameter *a* in the proposed DECOMPOSE method is the linear coefficient between R_2_* and a single-source volume susceptibility χ. The theoretically calculated linear coefficient *a* is 107.8 Hz/ppm/T in the static dephasing regime. The linear slope of ${\text{R}}_{2,+,-}^{*}\sim \chi_{+,-}$ is estimated to be 334 Hz/ppm for the Fe_2_O_3_ phantom and 371 Hz/ppm for the CaCO_3_ phantom (Fig. 4C&F); both are consistent with the theoretical value of 323.5 Hz/ppm at the static dephasing regime. However, this parameter *a* shall not be confused with the regression coefficient between R_2_* and volume QSM where the voxel contains a mixture of various susceptibility sources or when the static dephasing regime no longer holds. For example, even though the static dephasing regime assumption still holds as in the calibration phantoms, in the mixture phantom, the linear coefficient between R_2_* and QSM values is only 117.9 Hz/ppm (Fig. S3), significantly smaller than the theoretic value of 334 Hz/ppm, because the voxel contains a mixture of both Fe_2_O_3_ and CaCO_3_. Further, in brain tissues in vivo, because the effect of water diffusion can longer be neglected, the static dephasing regime no longer holds. Combining the motion narrowing effect which reduces R_2_* (Bloch, 1954) and the mixture of susceptibility sources in the brain, the coefficient between R_2_* vs QSM is expected to deviate from the static dephasing theory. For example, a previously study has reported the R_2_* vs QSM fitting result of 366 Hz/ppm at 7T (Deistung et al., 2013a) and 126.7 Hz/ppm at 3T (Li et al., 2014) both deviate from the static-dephasing-regime theoretic value of 754.8 Hz/ppm and 323.5 Hz/ppm respectively.

As to the susceptibility source’s geometry influence on the linear coefficient of ${\text{R}}_{2}^{*}$ vs *χ*, the value of 323.5 Hz/ppm is obtained for spherical susceptibility sources. At another extreme case, if parallel cylindrical susceptibility sources are considered, the coefficient becomes $a_{∥}=\frac{1}{2}\gamma B_{0}si{\text{n}}^{2}\theta$, where *θ* is the angle between parallel cylinders’ long axes and *B*_0_ field direction (Yablonskiy and Haacke, 1994). The maximum value of the coefficient is then 401.3 Hz/ppm at 3T. Being spherical and cylindrical are two extreme geometries of susceptibility sources. The linear coefficient of other geometries should be in between these two extreme values. More generally, the effective parameter *a* can be written as *a*_*e*ff_ = *a*_angle independent_ + *a*_angle dependent_(θ) with the orientation correction term accounting for geometric effect and susceptibility anisotropy. According to orientation-dependent T_2_* studies (Lee et al., 2011; Oh et al., 2013), the maximum variation of the R_2_* relaxation rate between 0 ~ *π* rotating angles is 8Hz in the corpus callosum region at 7T. The susceptibility magnitude of white matter lipid is larger than 0.1 ppm (Li et al., 2012; Lounila et al., 1994; Wharton and Bowtell, 2012), therefore, the maximum angel correction to the parameter *a* is less than 80 Hz/ppm, or 12 Hz/ppm/T. As shown in Fig. S10, within the range of *a* = 323.5 ~ 401.3 Hz/ppm, the resultant composite maps (PCS and DCS) are insensitive to different combinations of the linear coefficients *a*_+_, and *a*_−_. Numerically, the different combinations of coefficient result in a standard deviation of 0.7 ppb for PCS maps and 1.1 ppb for DCS maps which are negligibly small. Alternatively, one may adjust the parameter *a* for each voxel to account for the underlying geometries, however, this will either require a prior knowledge or increase the number of unknowns. Current model and solver use solely multi-echo gradient echo data as conventional QSM scans.

As discussed in 5.1, even with a perfect choice of *a*, the estimation of *χ* can be erroneous sometimes when the concentration is below 0.1. This effect is due to the difficulty of fitting for highly nonlinear parameters that lack unique solutions. It is therefore more advantageous to use the effective QSM, PCS and DCS, as they are robust against the estimation error of *χ* and the parameter choices of *a*.

### 5.4. Ex vivo temperature-dependence validation

One big challenge in performing the temperature-dependence analysis was the lack of an absolute reference for QSM. As temperature changes, the absolute bulk susceptibility of the whole sample changes. Such a change is not captured by QSM. Larmor frequency shift and phase pre-processing employed in QSM reconstruction methods remove the zero and first-order information of the phase, which leads to the QSM being referenced to the mean of the whole sample with STAR-QSM. As the temperature decreases, an increase of the paramagnetic susceptibility within the sample will cause an increase of total bulk susceptibility of the sample. Because STAR-QSM is referenced to the mean susceptibility, this increase will thus lead to an underestimation of the paramagnetic susceptibility and an apparent overestimation of the diamagnetic susceptibility whereas the physical diamagnetic susceptibility ought to remain stable with temperature changes. To address this issue, we used $R_{2}^{*}$ variation to identify the regions with minimum dependence on temperature and use those regions as QSM reference. Even so, slight temperature dependent changes in DCS may still be observed especially with the 5-echo data (Fig. 5 and 6), but less dramatic than that of PCS. There are debates about if formalin fixation will alter the measured QSM value (Birkl et al., 2016; C. Liu et al., 2011). Our ex vivo studies used only fixed tissues. Any potential alteration does not affect the conclusions as long as the paramagnetic source (i.e., iron in this case) remains paramagnetic.

The volume fraction of the three compartments (*C*_+_, *C*_−_, *C*_0_) also showed a slight temperature dependance. We estimated that the thermal expansion resulting from a 20-degree-Celsius range will lead to approximately 0.5% of volume change (if extracellular fluid is considered to have volumetric coefficient close to water and up to 1.5% if proteins/lipids and other biomolecules are considered (Frauenfelder et al., 1987; Rabin and Plitz, 2005). This is comparable to what we have observed. Nonetheless, the estimation of *C*_+_,_−_ parameters may be also corrupted by noise and QSM inaccuracies. Thus, caution is warranted in interpreting the results in this case.

### 5.5. Potential applications

Brain structures: PCS maps in Fig. 8, S7 and S9 showed clear traces of veins and small paramagnetic clusters which are disguised by diamagnetic component in conventional QSM. The DCS maps on healthy subjects’ images showed more complete white matter tracks than threshold QSM. The *C*_0_ maps corresponding to the low susceptibility fluid reveals clear delineation of the subthalamic nuclei (Deistung et al., 2013a; Li et al., 2020; Zhang et al., 2018). These parameter maps could provide a new tool to study brain structures and to understand brain development with longitudinal dataset.

Neurodegenerative diseases: In Fig. 9, statistical significance of PCS in different regions of brain are in good agreement with QSM reported in previous studies. Iron has been reported to be involved in neurodegenerative diseases (Barbosa et al., 2015; Bousejra-ElGarah et al., 2011; Jellinger, 1999; Ndayisaba et al., 2019). In the case of AD, iron overload is known to facilitate the aggregation of tau-protein and beta-amyloid. Although a previous study has reported iron oxidation state dependency of QSM and $R_{2}^{*}$ (Birkl et al., 2020). Specifically, according to their report, R2* decreased about 2 Hz in both white matter and the cortex when ferric iron is reduced to ferrous iron. For the current setting of the DECOMPOSE-QSM model, such subtle variations from oxidation state dependency are unlikely to be resolved. With the proposed DECOMPOSE-QSM, it is worth investigating if iron deposition and protein aggregation can be separated to assist the characterization of the underlying pathology.

Demyelination diseases: The ability of DECOMPOSE-QSM to separate sub-voxel paramagnetic and diamagnetic susceptibility maybe useful for imaging demyelination diseases such as multiple sclerosis (MS). Demyelination and iron accumulation can both occur in MS lesions which cannot be differentiated by QSM (Hametner et al., 2013; Mehta et al., 2013; Wisnieff et al., 2015). This issue may be addressed with DECOMPOSE-QSM.

Susceptibility anisotropy: The model is compatible with susceptibility anisotropy in white matter (Li et al., 2017; Wharton and Bowtell, 2015). While DCS is expected to be anisotropic in white matter, PCS is expected to be isotropic. By separating out the anisotropic component, DCS may be beneficial to improve the estimation of susceptibility tensor.

### 5.6. Limitations and future work

The proposed 3-pool model signal equation is highly simplified with the assumptions of susceptibility source being spherical and relaxation following a theory at the static dephasing regime. Further improvement of the method may incorporate the variations of susceptibility source geometries and the effect of diffusion.

The algorithm relies on an accurate echo-time dependent QSM input. If the QSM input is inaccurate, the algorithm will have an inaccurate phase to work with, then the resulting maps can be confounding. The interpretations of the individual parameters and further improving the accuracies of *χ*_+_, *χ*_−_ at low concentration level are still under investigation. Despite that, it is noteworthy that the composite maps (PCS and DCS) are highly accurate based on simulation and phantom experiments; the maps also enhance the contrast for paramagnetic component and diamagnetic components. For the in vivo PD vs. controls study, DCS showed significance difference in multiple regions in addition to PCS differences. The differences of DCS between the two groups may suggest changes in myelination. However, further studies including ex vivo validation are needed.

## 6. Conclusions and outlooks

The proposed DECOMPOSE-QSM algorithm utilizes both the phase and magnitude information to separate susceptibility components within a voxel. The model consists of three pools of susceptibility sources that are spatially distributed: zero susceptibility (reference susceptibility), diamagnetic and paramagnetic susceptibility. We engineered a novel cost function and developed an effective optimization routine to solve the challenging nonlinear inverse problem. The validity and the accuracy of the solutions was demonstrated with extensive simulation, phantom experiments, ex vivo experiments and in vivo brain scans. DECOMPOSE-QSM provides six parameters and composite susceptibility maps (PCS and DCS) to characterize the susceptibility compartments. We plan to further validate the method and individual maps with independent measures and test its applications.

## Supplementary Material

Supplementary Material

## Appendix

### Transverse relaxation rate at the static dephasing regime

The linear relationship between $R_{2}^{*}$ and susceptibility is observed in vivo (Wu et al., 2012a), and ex vivo (Bagnato et al., 2018; Hankins et al., 2009). The relaxation theory of MR signal behavior at the static dephasing regime provides the quantification of this linearity as follows (Brown, 1961; Yablonskiy and Haacke, 1994),

(A1) $$R_{2}^{\prime }=\frac{2\pi }{9\sqrt{3}}\mu_{0}\gamma M\delta =\frac{2\pi }{9\sqrt{3}}\gamma B_{0}\chi ,$$

where *B*_0_ is the external static magnetic field, μ_0_ is the vacuum magnetic permeability, M is magnetization (referenced to neutral medium), γ is the gyromagnetic ratio, δ is the volume fraction of the particle of the susceptibility sources, and χ is the measurable volume susceptibility corresponding to the values in QSM.

The size of susceptibility source affects the applicability of this theory (Bowen et al., 2002). Specifically, the theory of static dephasing fits better in cell settings than in nano particle settings. In our proposed model, susceptibility sources are treated as clusters of particles or molecules rather than individual particles or molecules of ions or lipids, which is a suitable application of the theory of transverse relaxation at the static dephasing regime, thus

(A2) $$R_{2}^{*}=R_{2}^{\prime }+R_{2}=\frac{2\pi }{9\sqrt{3}}\gamma B_{0}|\chi |+{\text{R}}_{2}.$$

The transverse relaxation rate *R*_2_, resulting from spin-spin interaction, is a function of local field shift, diffusion, and other intrinsic processes. The theoretical quantification of such can be derived using quantum mechanics with some proper approximations (Bloembergen and Morgan, 1961; Koenig and Kellar, 1995; Roch et al., 1999). At the current situation, this value is being treated as a parameter to be estimated rather than pre-measured or pre-analyzed.

### Magnetic field of a uniformly magnetized sphere

The source of the magnetics susceptibility is modeled as a uniformly magnetized sphere. The field of the interior of a sphere with uniformly distributed susceptibility χ situating in static magnetic field with strength of *B*_0_ is

(A3) $$B_{z,in}=\frac{2}{3}\mu_{0}M_{0}=\frac{2}{3}\chi B_{0}$$

while the field (at location *r*) outside such a sphere (with radius of *R*_0_, locating at *r*_0_) is equivalent to a dipole field,

(A4) $$B_{z,out\;}\left(\theta ,r,r_{0}\right)=\frac{\chi B_{0}{\text{R}}_{0}^{3}}{3{\left(r-r_{0}\right)}^{3}}\left(3{\text{cos}}^{2}\theta -1\right).$$

## Abbreviations:

QSM: Quantitative Susceptibility Mapping
DECOMPOSE-QSM: DiamagnEtic COMponent and Paramagnetic cOmponent SEparation of QSM
PCS: Paramagnetic Component Susceptibility
DCS: Diamagnetic Component Susceptibility
