# Supplementary material for: Decompose quantitative susceptibility mapping (QSM) to sub-voxel diamagnetic and paramagnetic components based on gradient-echo MRI data
Source: Neuroimage. Author manuscript; Available in PMC 2022 Jan 1. (PMC8720043; doi:10.1016/j.neuroimage.2021.118477)
Supplement: Supplementary Material [file NIHMS1762713-supplement-Supplementary_Material.pdf]

Supporting Materials

	Calibration Phantom 1			Calibration Phantom 2		
**ROI**	Concentration of Fe_2O_3 (mM)	Volume (mL) of 2 mM Fe_2O_3 (per 20 mL)	Measured susceptibility (ppm)	Concentration of Ca_2CO_3 (M)	Mass (g) of Ca_2CO_3 (per 20 mL)	Measured susceptibility (ppm)
**1**	0.00	0.0	0.002±0.006	0.00	0	-0.004±0.002
**2**	0.50	5.0	0.059±0.017	0.50	1.001	-0.032±0.011
**3**	0.75	7.5	0.089±0.016	0.75	1.501	-0.040±0.006
**4**	1.00	10.0	0.140±0.016	1.00	2.002	-0.055±0.019
**5**	1.25	12.5	0.162±0.018	1.25	2.502	-0.069±0.017
**6**	1.50	15.0	0.186±0.028	1.50	3.003	-0.073±0.018
**7**	1.75	17.5	0.209±0.031	1.75	3.503	-0.094±0.011

Table S1

Composition of calibration phantoms. The volume of each cylindrical ROI is 20 mL. Fe_2O_3 agarose solution for each ROI is prepared from diluting 2 mM concentrated Fe_2O_3 agarose solution with 1% agarose solution. Ca_2CO_3 agarose solution for each ROI is prepared directly by the weight of Ca_2CO_3 powder. The susceptibility is measured from STAR-QSM reconstruction. Two calibration phantoms were made to calibrate for the measurable ground truth volume susceptibility.

	Mixture Phantom					
**ROI number**	Ratio of Fe_2O_3	Ratio of Ca_2CO_3	Volume (mL) of 1.0 mM Fe_2O_3	Volume (mL) of 1.5 M $CaCO_3$	Measured susceptibility (ppm)	Predicted susceptibility (ppm)
**1**	0.50	0.50	10 mL	10 mL	0.044±0.013	0.034
**2**	0.00	1.00	0 mL	20 mL	-0.074±0.014	-0.073
**3**	0.15	0.85	3 mL	17 mL	-0.046±0.022	-0.041
**4**	0.35	0.65	7 mL	13 mL	0.008±0.012	-0.001
**5**	1.00	0.00	20 mL	0 mL	0.146±0.021	0.140
**6**	0.85	0.15	17 mL	3 mL	0.121±0.023	0.108
**7**	0.65	0.35	13 mL	7 mL	0.086±0.018	0.066

Table S2

Composition of susceptibility mixture phantoms. The volume of each cylinder ROI is 20 mL. 1.0 mM Fe_2O_3 agarose solution and 1.5 M Ca_2CO_3 agarose solution are prepared and then mixed with different ratio. The susceptibility is measured from STAR-QSM reconstruction. The predicted susceptibilities are based on calibration phantom results.

Maps	Regions	Normal Controls	PD patients	p value
QSM	CN	0.022(0.012)	0.023((0.014)	0.48
	RN	0.056(0.023)	0.080(0.033)	0.033*
	SN	0.060(0.012)	0.097(0.040)	0.005**
	GP	0.089(0.014)	0.096(0.018)	0.18
	PU	0.056(0.017)	0.051(0.022)	0.31
	Thal	-0.004(0.0004)	0.0005(0.002)	0.009**
PCS	CN	0.035(0.012)	0.031(0.016)	0.24
	RN	0.071(0.020)	0.099(0.013)	0.001**
	SN	0.075(0.013)	0.109(0.010)	0.000002**
	GP	0.097(0.006)	0.105(0.008)	0.014*
	PU	0.066(0.014)	0.058(0.022)	0.185
	Thal	0.014(0.003)	0.015(0.002)	0.20
DCS	CN	-0.004(0.002)	-0.003(0.002)	0.082
	RN	-0.005(0.003)	-0.002(0.001)	0.007**
	SN	-0.004(0.001)	-0.002(0.001)	0.004**
	GP	-0.003(0.001)	-0.002(0.001)	0.085
	PU	-0.003(0.001)	-0.002(0.001)	0.033*
	Thal	-0.012(0.002)	-0.010(0.001)	0.004**

Table S3

ROI analysis of PD vs controls for QSM, PCS, and DCS. Susceptibility values are in ppm with standard deviation presented in the parentheses. P values from one-tailed two sample t-tests are displayed in the last column. Symbols of “*” indicate significant difference: *p< 0.05, **p<0.01. CN: caudate nucleus; RN: red nucleus; SN: substantia nigra; GP: global pallidus; PU: putamen; Thal: thalamus.

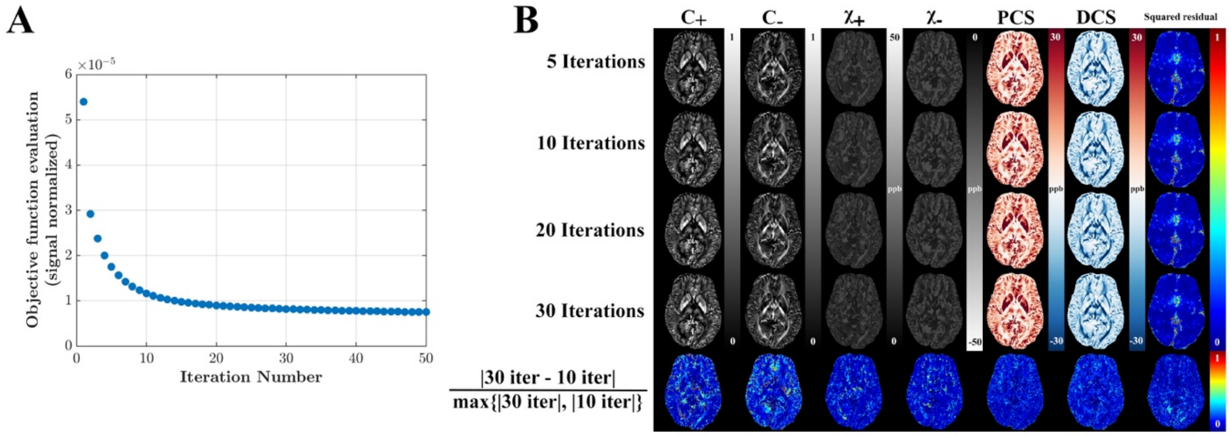

Figure S1

Examination of the solver's convergence

(A) Objective function value of a randomly chosen voxel normalized by signal intensity is dropping quickly within the first 10 iterations of alternating minimization. (B) Parameter maps and relative difference maps of in vivo experiments with 5, 10, 20, 30 iterations of alternating optimization procedure.

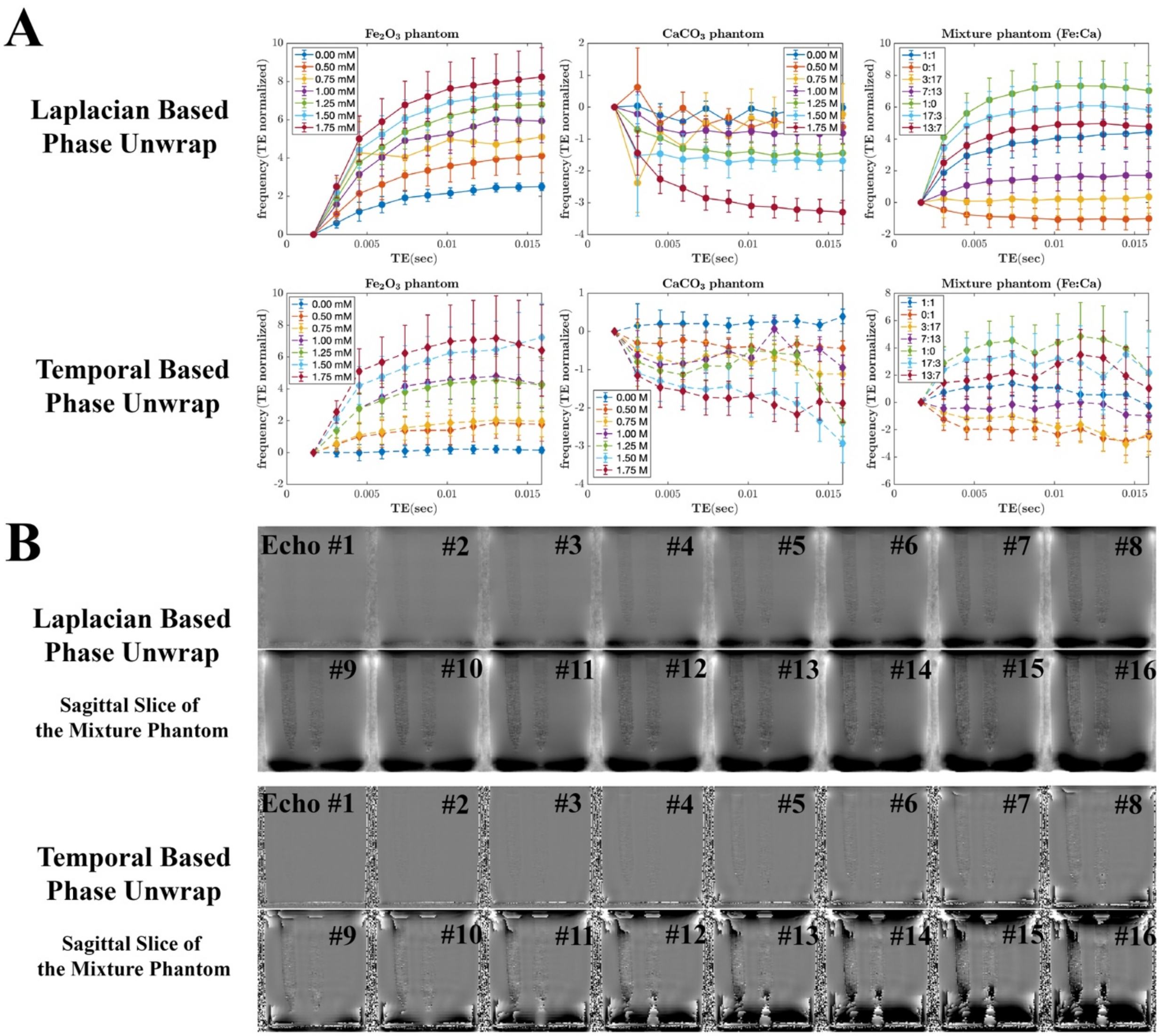

Figure S2

Comparison of unwrapped phase based on Laplacian (V-SHARP) method and temporal unwrapping method. (A) unwrapped phases relative to the first echo of each phantom over an ROI are plotted against the echo time from the first up to the eleventh echo. Echoes after the eleventh echo are discarded due to unreliable temporal-based phase unwrapping. (B) Unwrapped phase maps of one representative sagittal slice of the mixture phantom at each echo. Temporal based unwrapping fails when field inhomogeneity is too large or when SNR is too low.

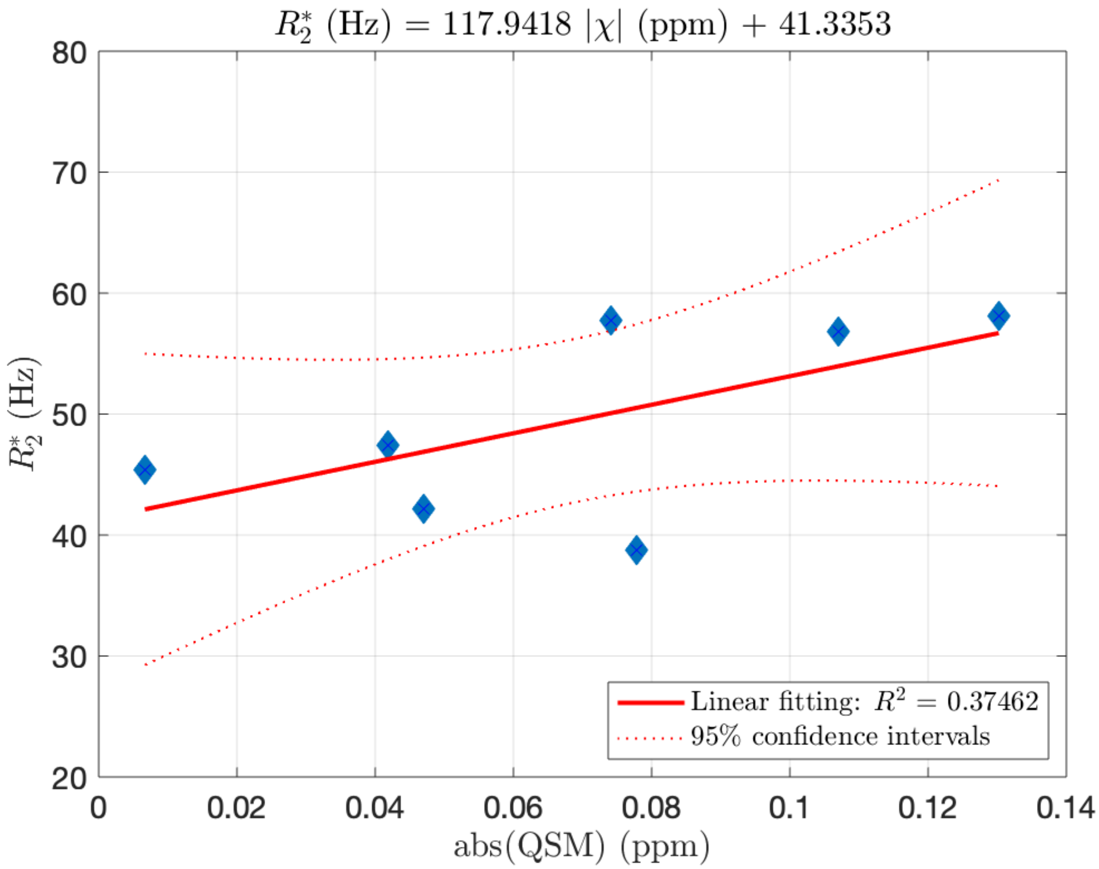

Figure S3

Linear fitting of measured R2* vs. the absolute value of QSM in the mixture phantom. The fitted linear coefficient is at 117.9 Hz/ppm, a much smaller value compared to the theoretical linear coefficient of R2* and tissue susceptibility χ.

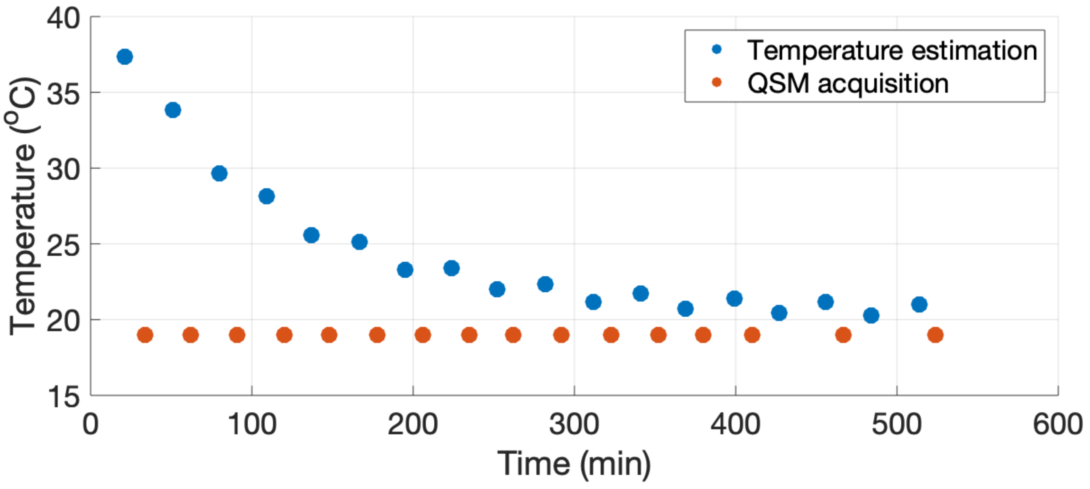

Figure S4

Temperature profile estimated from water proton spectrum before each QSM scan. The blue circle is the estimated temperature from calculating chemical shift. The orange circles indicate times of each QSM acquisition.

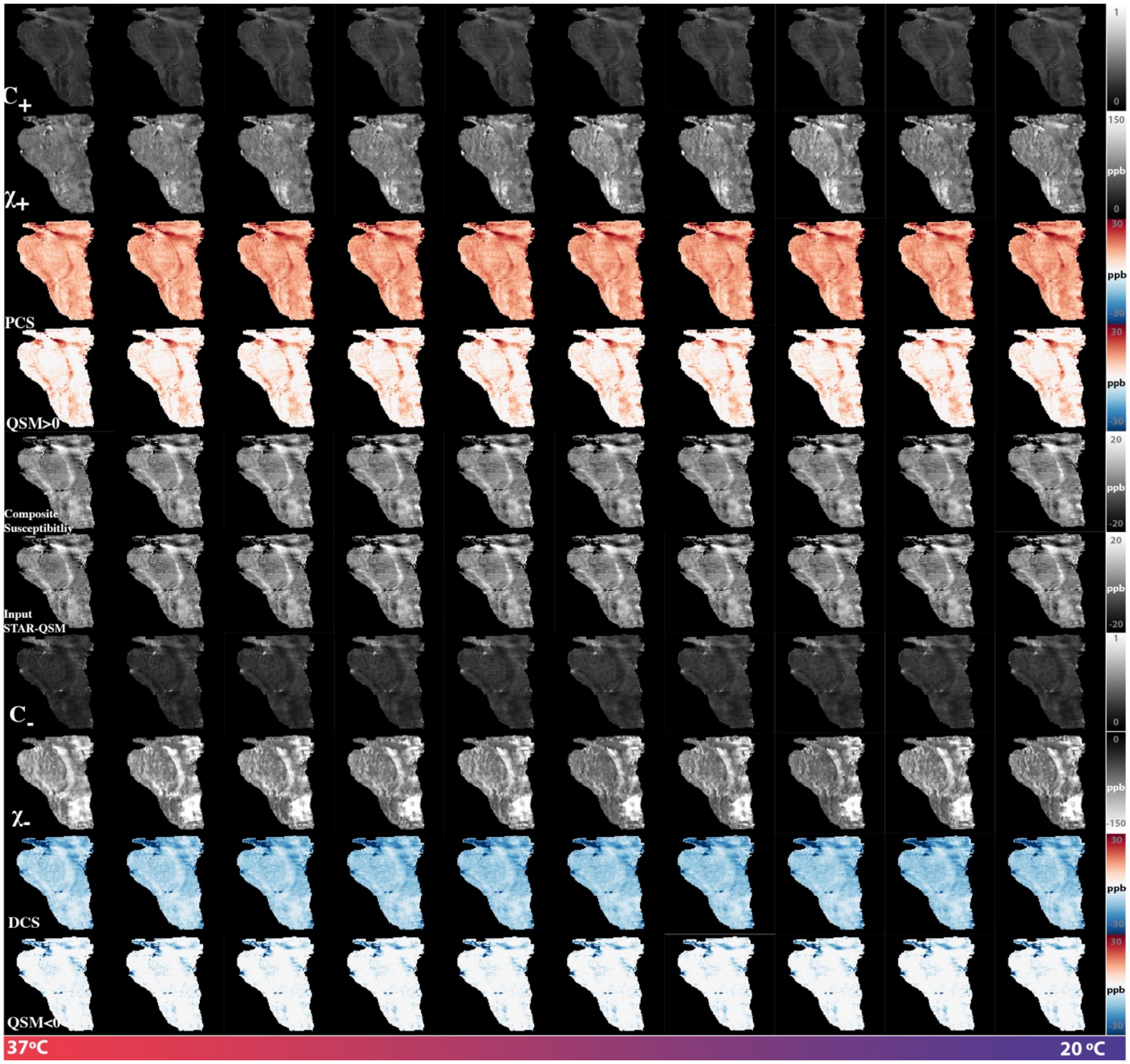

Figure S5

DECOMPOSE-QSM parameter maps of a brain stem specimen as a function of temperature. The increasing trend of PCS is visible, while the temperature-related change in DCS is minimal. The subplots relate to χ_- and DCS are displayed with inverted dynamic range to have a better visual contrast.

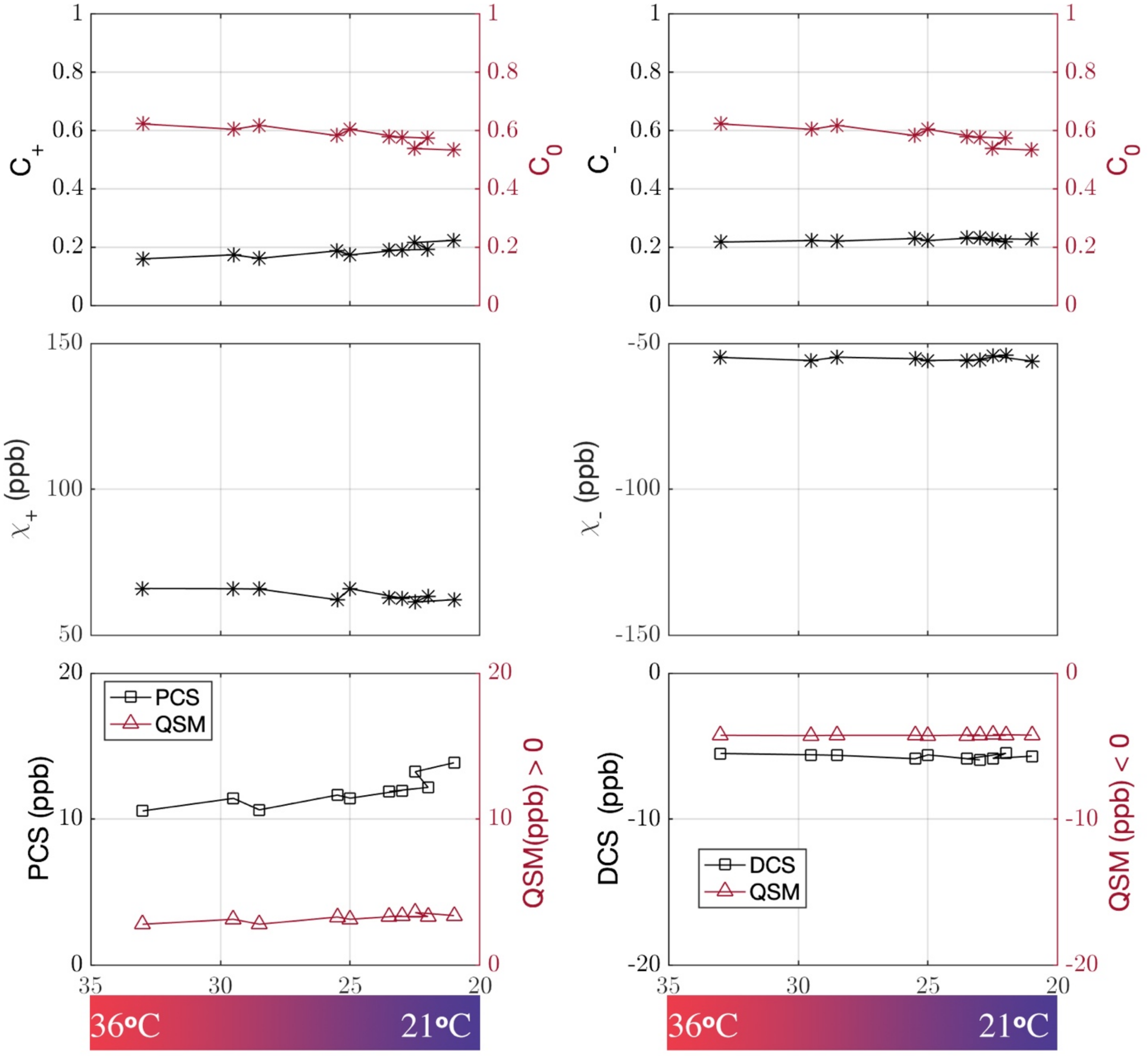

Figure S6

DECOMPOSE-QSM results of another brain stem specimen as a function of temperature. GRE data were acquired with twelve echoes. Temperatures range from 36 °C to 21 °C. Mean value is calculated from the non-zero mean of one representative slice. Note the paramagnetic component susceptibility is increasing with decreasing temperature as expected.

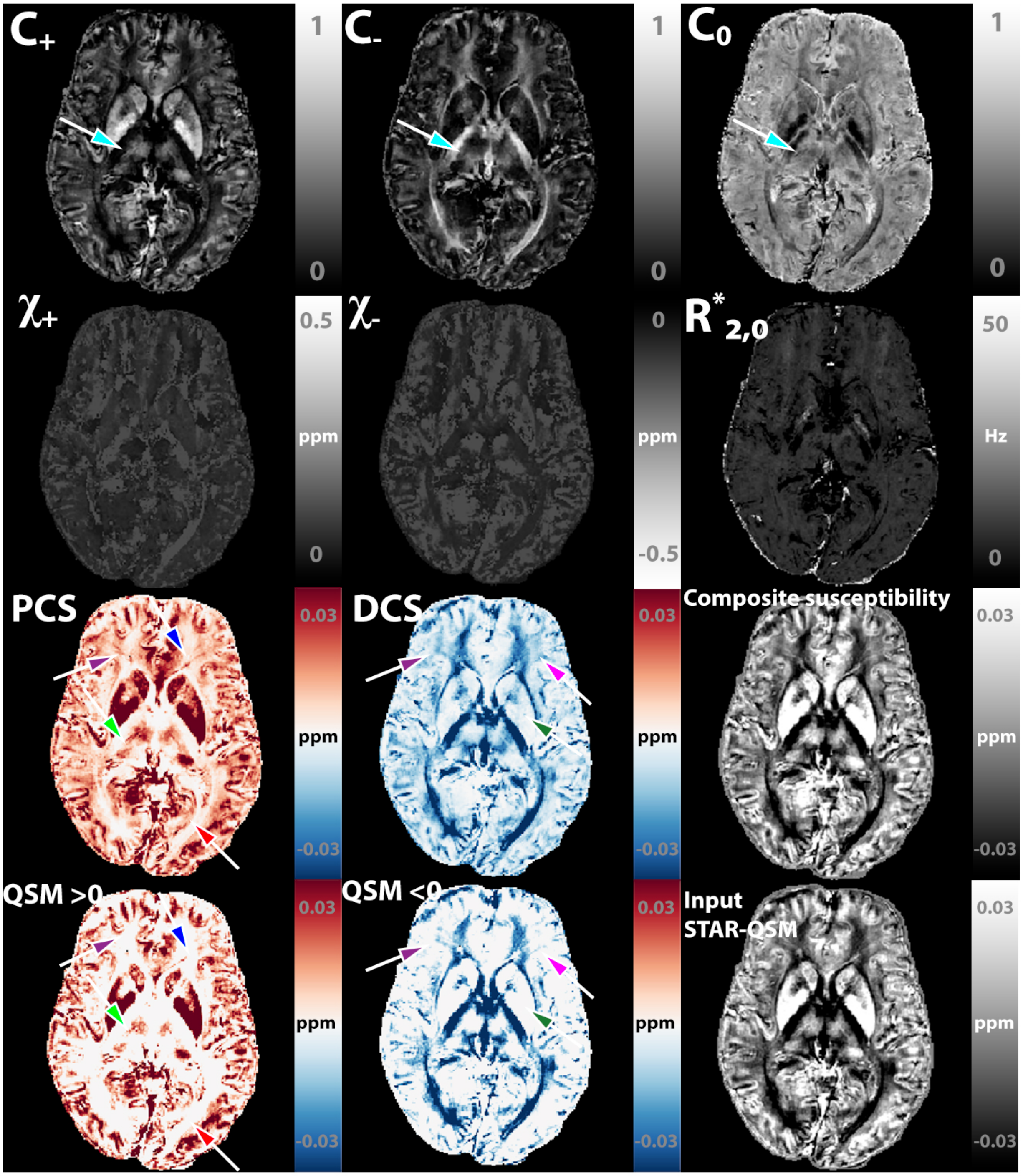

Figure S7

An additional illustration of DECOMPOSE-QSM being applied to a healthy adult study participant. Similar to Figure 8, first row: signal fraction maps show high fraction of paramagnetic susceptibility in gray matter, high fraction of diamagnetic susceptibility in white matter and high fraction of neutral component in the ventricles. The C_0 map particularly reveals clear delineation

of the subthalamic nuclei (arrow). Third and fourth row: The paramagnetic component susceptibility (PCS) and diamagnetic component susceptibility (DCS) show the existence of sub-voxel mixture of paramagnetic and diamagnetic components in both gray and white matter (arrows), which is not revealed in threshold QSM. The composite susceptibility is comparable to the input STAR-QSM. The subplots relate to the χ_- and DCS are displayed with inverted dynamic range to have a better visual contrast.

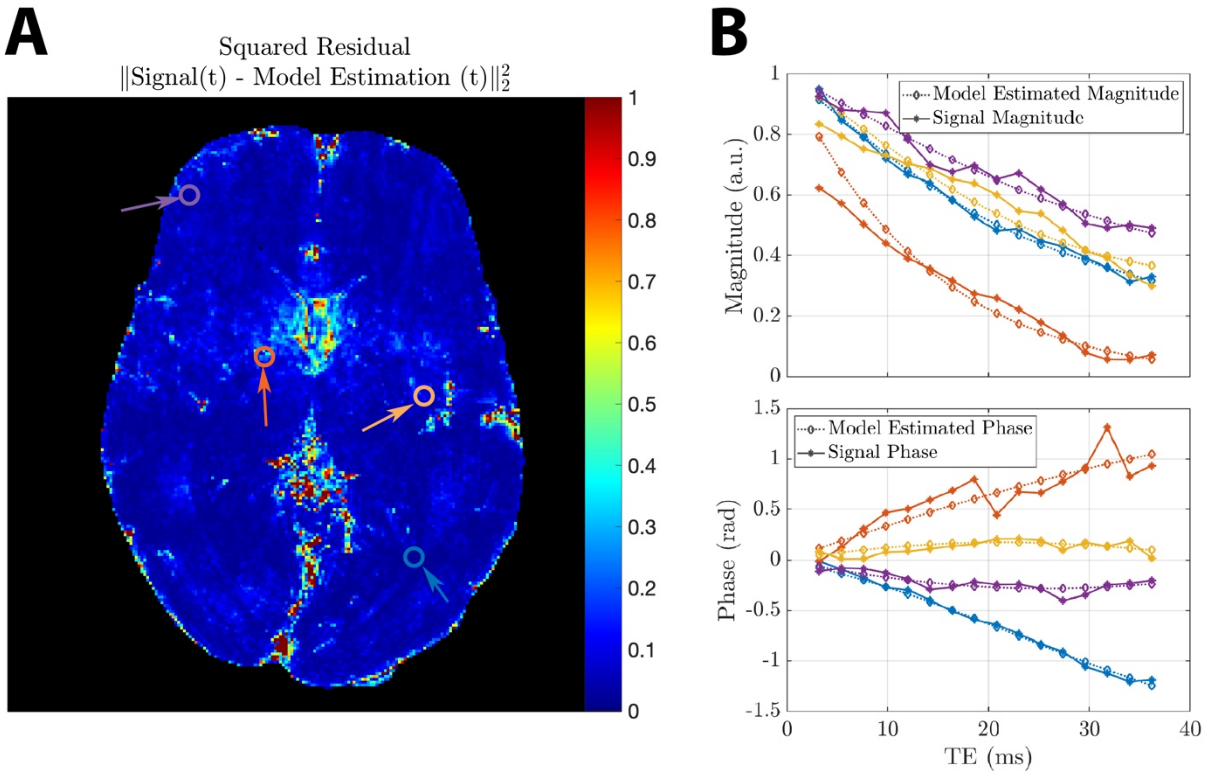

Figure S8

Model fitting performance for an *in vivo* case.

(A) Squared residual errors of a representative in vivo brain slice resulting from DECOMPOSE model fitting. (B) Magnitude and phase of four representative voxels in the illustrative brain slices. Solid curves are the input signal, dotted curves are the fitted curves.

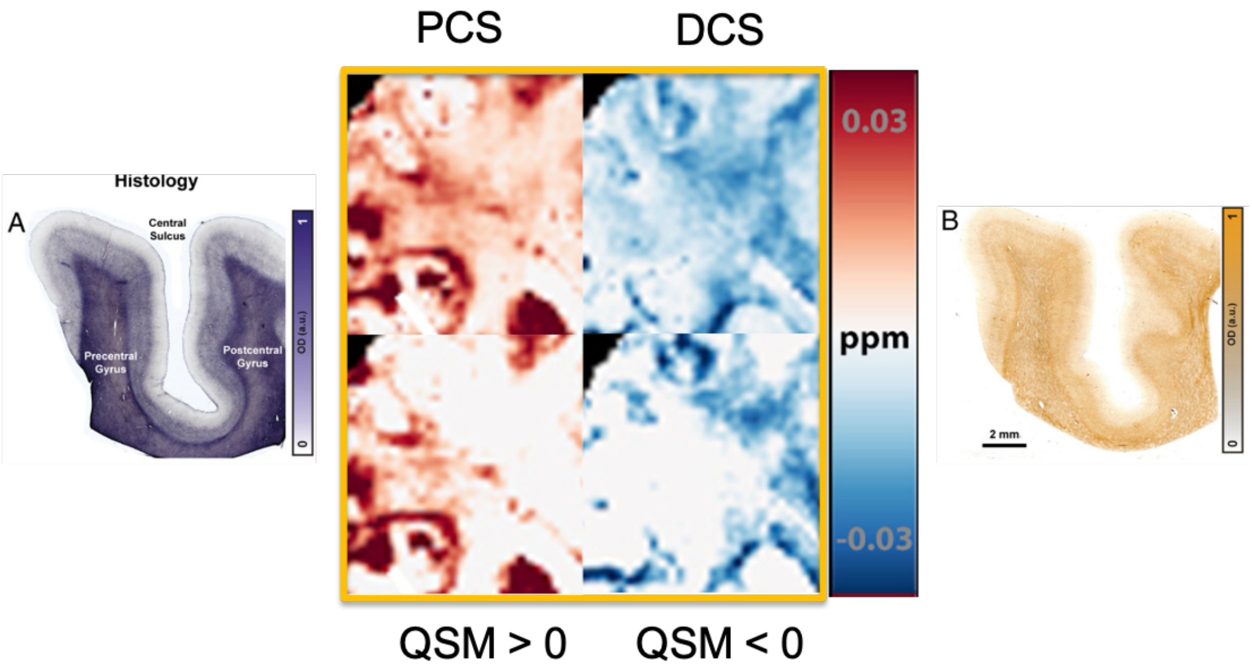

Figure S9

Visual comparison of previously reported histology and DECOMPOSE results. Photos A and B are histological staining of iron and myelin from (Stüber et al., 2014) respectively. Middle plot is the zoomed-in view of a cortical region of PCS and DCS (top) vs. threshold QSM (bottom). PCS matches the pattern of iron staining while DCS matches the myelin staining visually.

References

Stüber, C., Morawski, M., Schäfer, A., Labadie, C., Wähnert, M., Leuze, C., Streicher, M., Barapatre, N., Reimann, K., Geyer, S., Spemann, D., Turner, R., 2014. Myelin and iron concentration in the human brain: A quantitative study of MRI contrast. NeuroImage 93, 95–106. https://doi.org/10.1016/j.neuroimage.2014.02.026

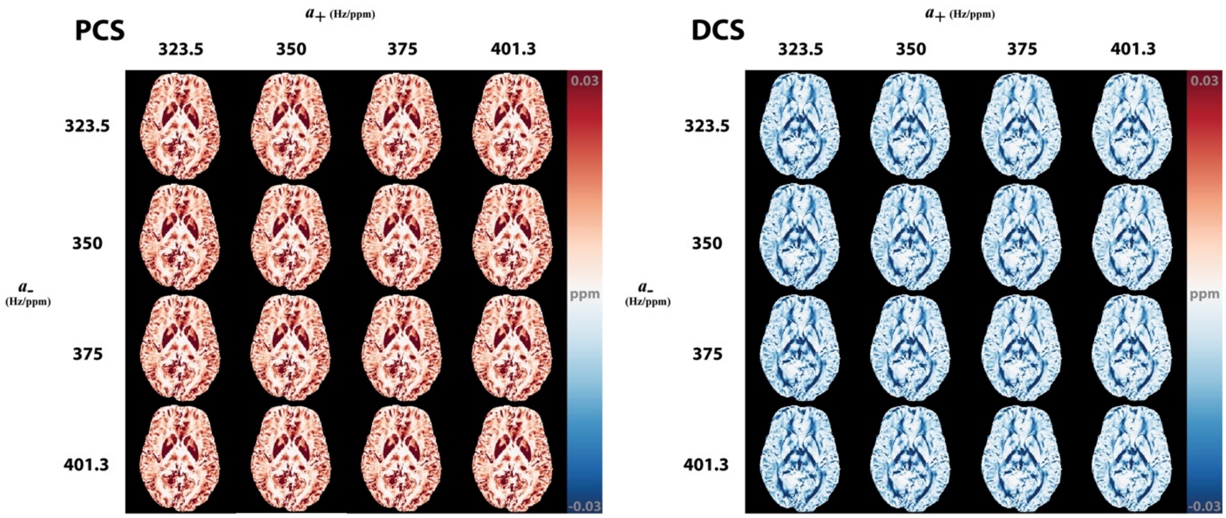

Figure S10

DECOMPOSE-QSM performed with different combination of choices of coefficient of linear relaxation relation with susceptibility. The linear coefficient depends on the shape of the susceptibility source. If the susceptibility source is considered to be spherical, the coefficient is estimated to be 323.5 Hz/ppm, whereas if the susceptibility source is considered to be parallel cylinders, the maximum value of the coefficient is 401.3 Hz/ppm. The true susceptibility source situation should be in between these two extremes. The illustration shows that within the range, the standard deviation of the resulting PCS and DCS is 0.7 ppb and 1.1 ppb respectively; both are negligibly small compared to experimental precision.

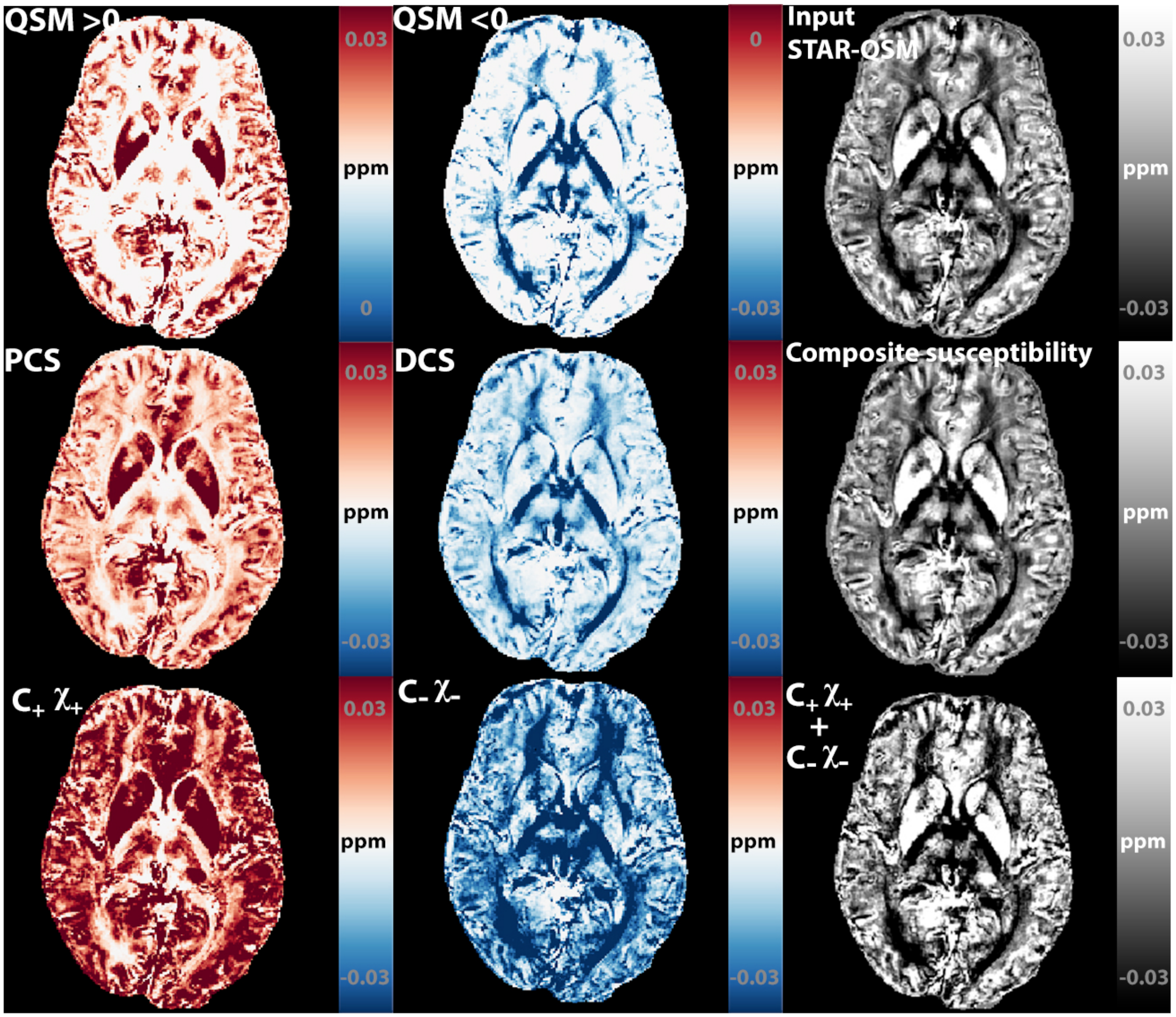

Figure S11

Different DECOMPOSE-QSM result representations are showing in row 2 and row 3 comparing to threshold QSM results in row 1. Paramagnetic component susceptibility (PCS) is calculated using estimated paramagnetic component related parameters (C_+, χ_+) and estimated parameters related to neutral component $(C_0, R^*_{2,0})$ according to equation 10. Diamagnetic component susceptibility (DCS) and composite susceptibility are calculated likewise according to equation 11 and 12. Plots in last row are direct multiplications of concentration C_+ (or C_-) and χ_+(or χ_-) and the superposition of both components' direct multiplications. The direct multiplication of each component reflects the true susceptibility without neutral component being considered hence the brighter appearance.
